# *GhCalS5* is involved in cotton response to aphid attack through mediating callose formation

**DOI:** 10.3389/fpls.2022.892630

**Published:** 2022-07-20

**Authors:** Natasha Isabel Tanatsiwa Mbiza, Zongwei Hu, Haoran Zhang, Yi Zhang, Xincheng Luo, Yuxue Wang, Yi Wang, Ting Liu, Jianping Li, Xiangping Wang, Jianmin Zhang, Yonghao Yu

**Affiliations:** ^1^College of Agriculture, Institute of Entomology, Yangtze University, Jingzhou, China; ^2^College of Life Sciences, Yangtze University, Jingzhou, China; ^3^Guangxi Key Laboratory of Biology for Crop Diseases and Insect Pests, Guangxi Academy of Agricultural Sciences, Nanning, China

**Keywords:** *GhCalS5*, cotton, callose, plant response, aphid resistance

## Abstract

Callose synthase plays an essential role in plant growth and development and in response to all sorts of stresses through regulating callose formation. However, few research about the function and mechanism of the insect resistance of callose synthase genes have been reported in cotton. In this study, a cotton callose synthase gene *GhCalS5* was cloned, and its function and mechanism of resistance to cotton aphids were analyzed. The expression of *GhCalS5* was significantly upregulated in both, leaves and stems of cotton plants at 48 h after cotton aphid infestation and in the leaves of cotton plants at 24 h after salicylic acid treatment. The overexpression of *GhCalS5* enhanced cotton resistance to cotton aphids. Expectedly silencing of *GhCalS5* reduced cotton resistance to cotton aphids. Overexpression of *GhCalS5* enhanced callose formation in cotton leaves. Our results suggest that *GhCalS5* is involved in cotton resistance against cotton aphids by influencing callose formation.

## Introduction

Callose is a β-1, 3-glucan polymer synthesized by callose synthases in higher plants (Wang et al., 2021). It exists in multiple places in plant cells. For example, callose is transiently deposited in the cell plate at the cytokinesis stage and is a vital component of the transient cell wall surrounding pollen mother cells, the four microspores after meiosis, and the pre-cell wall of the growing pollen tube tip (Mccormick, 1993; Hong et al., 2001). Callose is also present in sieve plates under normal plant growth and developmental conditions but can accumulate quickly and plugs the sieve pores under stress (Xie et al., 2011). More importantly, callose is deposited between the plasma membrane and the pre-existing cell wall at sites of pathogen attack (Voigt, 2014). In addition, callose is also synthesized in the neck region of plasmodesmata during abiotic and biotic stresses (Zavaliev et al., 2011). Callose synthase (CalS) is also referred to as glucan synthase-like (GLS; Richmond and Somerville, 2000; Verma and Hong, 2001). Twelve *AtCalSs* in *Arabidopsis thaliana* have been described (Hong et al., 2001), as well as 12 *CsCalSs* in *Citrus sinensis* (Granato et al., 2019), 32 *BnCalSs* in *Brassica napus* (Liu et al., 2018), 8 *VvCalSs* in *Vitis vinifera* (Yu et al., 2016), 7 *HvCalSs* in *Hordeum vulgare* (Schober et al., 2009), 15 *BraCalSs* in Chinese cabbage (Pu et al., 2019), and 27, 28, 16, and 15 *GhCalSs,* respectively, in *Gossypium hirsutum*, *G. barbadense*, *G. raimondii*, and *G. arboreum* (Feng et al., 2021) have been reported.

Callose synthases play crucial roles in plants by regulating callose deposition. Firstly, it is involved in normal plant growth and development of plants. For example, callose synthase *GSL7* is necessary for inflorescence growth in *Arabidopsis* (Paul Barratt et al., 2011). A callose synthase *Tie-dyed2* mutant of *Zea mays* displays variegated green and yellow leaves (Slewinski et al., 2012). Secondly, callose synthases are also influenced by multiple abiotic stress. For instance, when maize leaf tip was stimulated by low temperature, action potential was generated in the phloem, intercellular movement from mesophyll to sheath cells decreased significantly, callose content in leaves increased, and the transport of photosynthates in phloem decreased (Fromm et al., 2013). *A. thaliana AtCalS7* (*AtGSL7*), *AtCalS8* (*AtGSL4*), and *AtCalS12* (*AtGSL5*/PMR4) are associated with callose synthesis upon mechanical wounding (Cui and Lee, 2016). Additionally, callose synthases are also affected by various types of biotic stress. Recently, callose synthase family genes have been found to play an essential role in citrus defense response to *Candidatus* Liberibacter asiaticus, and a novel demonstration of RNAi in citrus reveals the importance of citrus callose synthase in defense against *Xanthomonas citri subsp. citri* (Enrique et al., 2011; Granato et al., 2019). Transient expression of the *A. thaliana* callose synthase *PMR4* increases penetration resistance to powdery mildew in barley (Blümke et al., 2013), and this pathogen-induced callose deposition functions as a chemical and physical defense mechanism by reinforcing plant cell wall (Ellinger and Voigt, 2014). In addition to induction by plant disease invasion, rapid deposition of callose at the point of attempted penetration was observed after insect feeding (Hao et al., 2008; Kuśnierczyk et al., 2008) or nematode infestation (Hofmann et al., 2010). When attacked by piercing insects, plants activate callose deposition, which hinders phloem’s flow, discourages phloem feeding, and this is the key resistance mechanism in rice varieties resistant to brown planthopper (*Nilaparvata lugens*; Hao et al., 2008). An *AtCalS7* gene has been associated with the synthesis of callose in the sieve plates of phloem in response to stresses resulting in the phloem blockage (Paul Barratt et al., 2011; Xie et al., 2011). Aphids and silver whitefly nymphs have been reported to induce callose in plants upon infestation (Botha and Matsiliza, 2004; Kempema et al., 2007). Meanwhile, callose synthase is mediated by all signaling pathways, and different biological regulatory processes also involve hormones. For example, abscisic acid (ABA) increased rice callose synthase activity, promoted callose deposition, and improved its resistance to *N. lugens* (Liu et al., 2017). *Arabidopsis* callose synthase 1 (*CalS1*) and *CalS8* have been identified as the key genes involved in altering plasmodesmal permeability upon mechanical wounding stress (Dong et al., 2008). While previous studies have focused on pathogen-induced callose, little research has been reported on herbivore-induced callose.

Herbivore feeding accounts for > 20% of net plant productivity in vegetation systems, including the loss of foliage, sap, and root feeding (Agrawal, 2011). These losses occur despite increased pesticide use, highlighting the need to develop sustainable approaches for pest control with less reliance on chemical inputs. Cotton aphid (*Aphis gossypii*) is the most problematic pest to growers in cotton production. They insert their stylets into plant tissues and probe until they contact a phloem vessel to feed on phloem sap, resulting in the plant’s retarded growth, giving rise to deformed leaves, and the infestation may result in the death of young plants if it occurs early in the season. Survival of plants upon aphid attack depends on a dynamic defense system involving structural barriers (including plant callose deposition) and attraction of natural enemies of target pests in nature (Sánchez-Sánchez and Morquecho, 2017). Therefore, the cotton gene *GhCalS5* was cloned in this study, and the gene function and mechanism in cotton response to cotton aphids were determined through overexpressing and silencing methods, respectively. Our results will help reveal that the *GhCalS5* gene has a role in cotton plant response to aphid infestation.

## Materials and methods

### Plant materials, growth conditions, and aphid rearing

Red-leaf cotton (*G. hirsutum*), a cotton aphid-resistant variety, was provided by the Cotton Research Institute of Chinese Academy of Agricultural Sciences. The seeds of red-leaf cotton were planted directly in the nutritional soil (peat soil: perlite: vermiculite = 4:1:1) in 30-cm-diameter plastic pots. The plants were kept and grown in a growth room with 75% relative humidity and photoperiod 16 h light/8 h darkness at 25°C. Cotton aphids were collected from the laboratory cotton field at Yangtze University, cultured on Coker 312, a variety with weak resistance to aphids, and maintained in an environment chamber at 25°C with a long day photoperiod (16 h light and 8 h darkness). Active adult aphids of the same age and size were used in all the experiments.

### Sequence analysis of *GhCalS5*

The coding sequence (CDS) of *GhCalS5* from the leaf transcriptome data of red-leaf cotton induced by cotton aphids was amplified as a cotton cDNA template, using gene-specific primer pair for *GhCalS5* (Supplementary Table S1). The PCR products were linked to the pMD 18 T cloning vector (Takara) and transformed into *Escherichia coli* strain DH5α. Positive clones were inoculated for sequencing of *GhCalS5*. The obtained sequence was used as a query in a BLASTp program^1^ in the National Center for Biotechnology Information (NCBI), and the protein sequences with the highest homology to *GhCalS5* from other plant species were downloaded. The theoretical isoelectric point and molecular weight of *GhCalS5* protein were obtained using Lasergene 7.1 software. The alignment of callose synthase proteins from different species was carried out using the DNAMAN, and the phylogenetic tree was constructed by the software MEGA11 with the maximum likehood method. Numbers on the branches represent bootstrap values (1,000 replicates).

### Vector construction and transformation of cotton

The PCR products were first cloned into the pMD-18T cloning vector, and the intermediate vector pMD18-T-*GhCalS5* was then digested by Xba1 and BamH1. The resultant digestion fragment was further inserted into the corresponding sites of the overexpression vector pBI121 containing Cauliflower mosaic virus (CaMV) 35S promoter to generate pBI121-*GhCalS5.* The tobacco rattle virus (TRV)-based VIGS system was used to silence *GhCalS5* expression in cotton. The pMD-18T-*GhCalS5*-digested fragment was inserted in the corresponding sites in the plasmid pTRV2 to generate the virus gene-silencing vector TRV: *GhCalS5*. The plasmids of TRV1, TRV:00 (the negative control with empty vector), TRV: *GhCalS5*, pBI121 (the negative control with empty vector), and pBI121-*GhCalS5* were transformed into *Agrobacterium* strain GV3101 *via* the freeze–thaw method (Degefu and Hanif, 2003), respectively. Recombinant bacteria were separately grown overnight from pre-cultures at 28°C and 200 rpm in an orbital shaker using LB medium (supplemented with appropriate antibiotics), pelleted by centrifugation, and resuspended in MMA (10 mM MES, 10 mM MgCl_2_, and 100 mM acetosyringone; Norkunas et al., 2018). The GV3101 strain harboring TRV1 was mixed with those harboring TRV: *GhCalS5* at a 1:1 ratio. The mixture and three *A. tumefaciens* harboring TRV:00, pBI121-*GhCalS5*, or pBI121 were infiltrated into the cotyledons of 8-day-old cotton using a needless syringe, respectively.

### RNA extraction and quantitative RT-PCR

Total RNA was extracted from cotton roots, leaves, and stems using a Fast pure plant total RNA isolation kit (Vazyme, Nanjing, China) according to the manufacturer’s instructions. The quality of RNA was verified after separation on agarose gel by ethidium bromide staining and quantified using a NanoPhotometer N50 (Implen). 2 μg total RNA of each sample was reverse transcribed into cDNA using the PrimeScript^™^RT Reagent Kit (TaKaRa, Shiga, Japan) according to the manufacturer’s instructions. The cotton ubiquitin gene *GhUBI1* (accession number: EU604080) was used as the reference gene. The fold change of the relative genes was quantified using the 2^–∆∆CT^ method (Livak and Schmittgen, 2001). Each sample had three biological repeats, which were repeated three times.

### Expression patterns of *GhCalS5* under different treatments in cotton

In order to detect expression patterns of *GhCalS5* in cotton tissues upon aphid infestation, the tissue samples from cotton roots, stems, and leaves of cotton cotyledons of 14 days were harvested at 24, 48, and 72 h after aphid infestation or no-cotton aphid infestation. Cotton cotyledons of 14 days seedlings were sprayed with SA (5 mmol/l) solution as a foliar spray until runoff and collected at 24, 48, and 72 h post spray. Three independent tests with three biological replicates were performed.

### Host choice assay

The host choice test of cotton aphids was performed in 9-cm-diameter Petri dishes. The cotyledons were detached from *GhCalS5*-overexpressing, pBI121, and wild-type cotton plants, and their petioles were wrapped with a wet cotton ball. Each group of cotyledons was symmetrically placed on the inner edge of the Petri dish. A total of 20 apterous adults of cotton aphids were put at the center of the Petri dish and counted after 24, 48, and 72 h. The host no-choice assay was performed *in vivo*; 20 apterous adults of cotton aphids were inoculated on each of the 9-day-old *GhCalS5*-overexpressing, pBI121 (control), and wild-type cotton plants or 15-day-old *GhCalS5*-silenced plants-silenced, TRV:00, and wild-type cotton plants; and the number of aphids was counted at 24, 48, and 72 h or 7–22 days after feeding, respectively. Each treatment was replicated three times.

### Determination of aphid feeding activity

The feeding activity of cotton aphids can be determined by measuring the amount of honeydew produced. Split Whatman filter papers were placed under each plant on *GhCalS5*-overexpressing, pBI121, and wild-type cotton plants or *GhCalS5*-silenced plants, TRV: 00, and wild-type cotton plants; 20 apterous aphid adults were put on each plant. A plastic membrane was placed under the filter paper to avoid the absorbance of water from the soil. Whatman filter papers were used to collect honeydew after 24, 48, and 72 h for *GhCalS5*-overexpressing, pBI121, and wild-type cotton plants and 8 days, 15 days, and 22 days for *GhCalS5*-silenced plants, and TRV: 00, and wild-type cotton plants, respectively. Whatman filter papers were soaked in 0.1% (w/v) ninhydrin solution in acetone and dried in a 65°C oven for 30 min, and purple spots were shown when honeydew was stained by ninhydrin (Kim and Jander, 2007). To quantify the honeydew stains, stained filter papers were cut into pieces and extracted with 1 ml of 90% (v/v) methanol for 1 h at 4°C with continuous agitation. The supernatant absorbance was measured at 500 nm after centrifugation at 6,000× *g* for 1 min. 90% methanol was used as a blank. Each treatment was replicated four times.

### Cotton leaf callose analysis

Aniline blue was used to stain callose deposition, according to (Zhang et al., 2014). In short, the cotyledons leaves from *GhCalS5*-overexpressing, pBI121, and wild-type cotton plants and *GhCalS5*-silenced plants, TRV: 00, and wild-type cotton plants were put in 5 ml lactophenol solution: phenol, glycerol (100%), lactic acid, distilled water, and absolute ethanol (1:1:1:1:8 v/v), and then immersed by the vacuum pump. Leaves were incubated in a water bath at 65°C for 1 h. Cotton leaves were soaked in 0.01% aniline blue staining solution (0.15M K_2_HPO_4_, pH 9.5). Callose deposition was visualized under Nikon fluorescent microscopy equipped with an ultraviolet lamp (330–380 nm). Callose deposits were quantified by a protocol previously described by Jin and Mackey (2017). Each treatment was replicated four times.

### Statistical analysis

The data were obtained from three independent technical or biological replicates per treatment and presented as the mean ± standard error. All data were subjected to analysis of variance (ANOVA). Mean differences were estimated by Tukey’s honestly significant difference (HSD) using statistical software SPSS 26.0 (SPSS Inc., Chicago, IL, United States of America) and GraphPad Prism 8.0 software. Statistical significance was considered when the *p*-value was less than 0.05.

## Results

### *GhCalS5* sequence analysis

**Figure 1:**
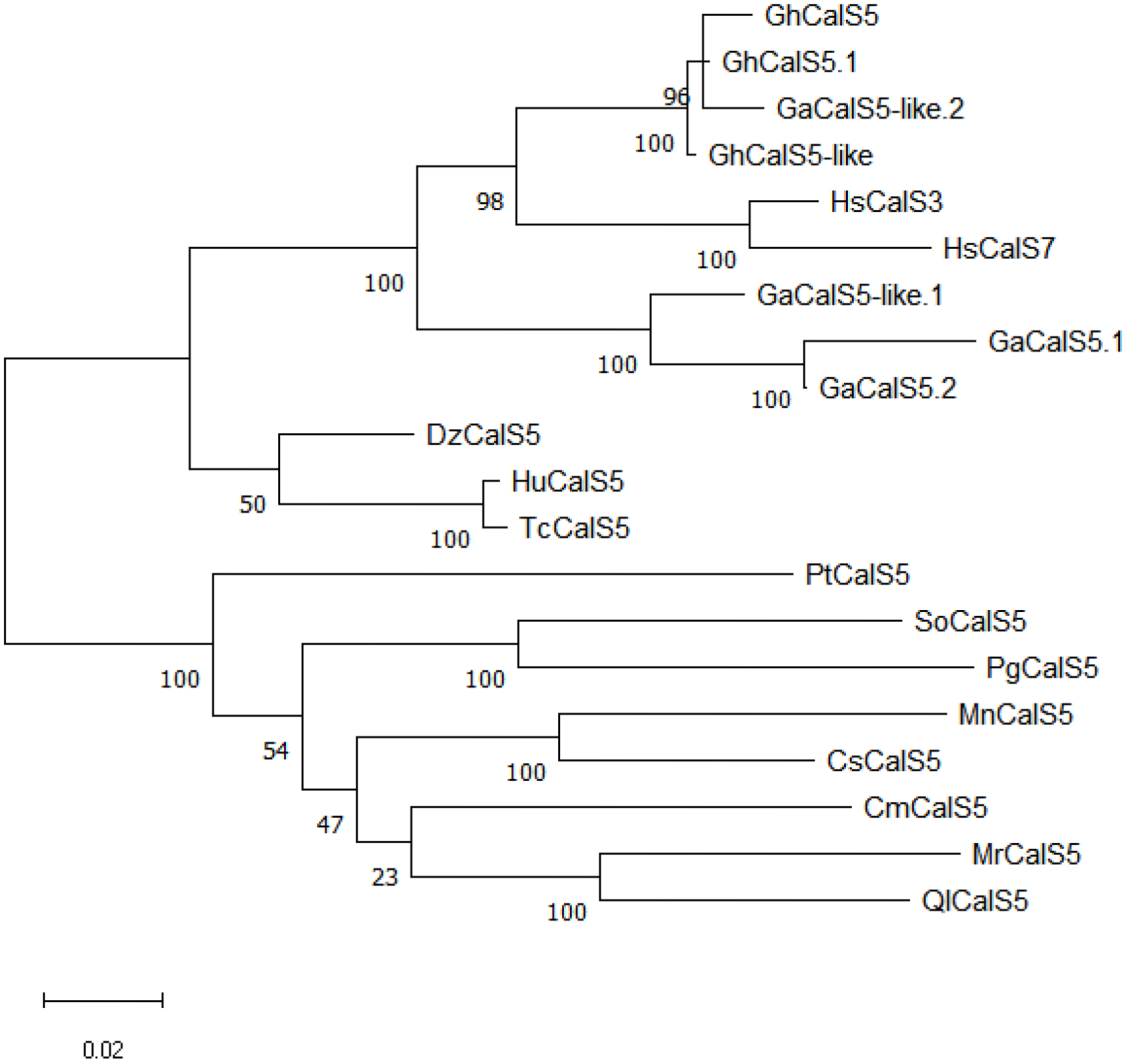
Phylogenetic analysis of *GhCalS5* protein and *CalS* proteins of other plant species. The phylogenetic tree was constructed by the maximum likehood method using MEGA11 software. The accession numbers of sequence data were as follows: *GhCalS5.1*, *Gossypium hirsutum CalS5.1* [XP_016676296.2]; *GaCalS5.1*, *Gossypium arboreum CalS5.1*[KHG21671.1]; *GaCalS5-like.1*, *Gossypium austral CalS5-like.1* [KAA3479749.1]; *GaCalS5.2*, *G. arboreum CalS5.2* [KHG21670.1]; *HsCalS3, Hibiscus syriacus CalS3*[KAE8662811.1]; *HsCalS7*, *H. syriacus CalS7* [KAE8680723.1]; *DzCalS5*, *Durio zibethinus CalS5* [XP_022750939.1]; *HuCalS5*, *Herrania umbratica CalS5* [XP_021300964.1]; *TcCalS5, Theobroma cacao CalS5* [EOX92541.1]; *MnCalS5*, *Morus notabilis CalS5* [EXB36810.1]; *SoCalS5*, *Syzygium oleosum CalS5*[XP_030440758.1]; *CsCalS5*, *Cannabis sativa CalS5*[XP_030499529.1]; *MrCalS5*, *Morella rubra CalS5*[KAB1210572.1]; *PgCalS5*, *Punica granatum CalS5*[XP_031399619.1]; *PtCalS5*, *Populus trichocarpa CalS5*[XP_024456702.1]; *CmCalS5, Cucurbita maxima CalS5*[XP_023007270.1]; *QlCalS5*, *Quercus lobata CalS5*[XP_030973950.1]; *GaCalS5-like.2*, *G. arboreum CalS5-like.2* [XP_017647032.1]; *GhCalS5-like*,*G. hirsutum CalS5-like*[XP_016698828.1].

The sequence analysis showed that *GhCalS5* CDS was made up of 1,851 bp. Its encoded protein comprises 616 amino acids, its relative molecular mass is 42.58 kDa, and its theoretical isoelectric point is 9.689 (Supplementary File S1). The maximum likehood method was used to analyze the phylogenetic relationships between *GhCalS5* and callose synthase from other plant species. From the phylogenetic tree, the amino acid sequence of *GhCalS5* was located in a clade where *GhCalS5.1* and *GaCalS5-like.2*, and *GhCalS5-like*, were tightly clustered. Other homologous proteins were clustered into other large clusters (Figure 1). The close relationship between *GhCalS5*, *GhCalS5.1*, *GaCalS5-like.2*, and *GhCalS5-like* suggests having a similar function (Supplementary Figure S1).

### *GhCalS5* expression is induced by aphid and SA

**Figure 2:**
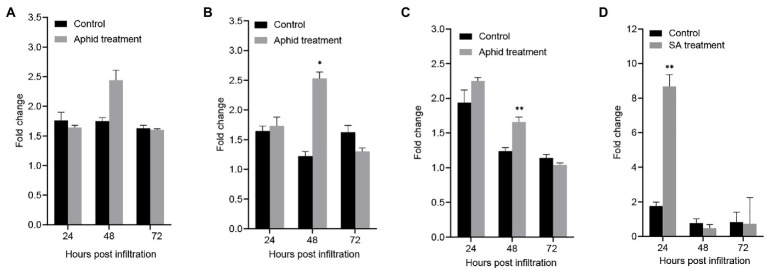
Expression of *GhCalS5* in cotton plants after cotton aphid treatment and after SA treatment. **(A)** Root after aphid treatment, **(B)** stem after aphid treatment, **(C)** leaf after aphid treatment **(D)** leaf after SA treatment. Error bars represent the standard deviation of measurements from three biological replicates. Number of plants used = 3 per treatment. Independent Student’s *t*-tests analyzed data. Error bars represent the SD of three biological replicates. Statistical significance for treatment effects is marked (^*^p < 0.05) or (^**^p < 0.01).

The qPCR results showed that *GhCalS5* was expressed in root, stem, and leaves of *G. hirsutum* after cotton aphid feeding, salicylic acid (SA) treatment, or no-treatment (control; Figure 2). Compared with control, no significance was observed in the expression level of *GhCalS5* in root at 24, 48, and 72 h after aphid feeding (Figure 2A). The *GhCalS5* transcript was significantly upregulated at 48 h after aphid feeding in stems. However, no significant difference was detected in the expression level at 24 and 72 h between cotton aphid and no-aphid feeding (Figure 2B). The *GhCalS5* expression level was significantly upregulated at 48 h after aphid feeding in leaves, but no significant difference was detected at 24 and 72 h between cotton aphid feeding treatment and control (Figure 2C). In addition, the expression level of *GhCalS5* was very significantly upregulated in the leaves at 24 h after SA treatment, but no significant differences were detected at 48 and 72 h between SA treatment and control (Figure 2D). These results suggested that cotton aphid feeding or SA treatment could induce a transient upregulation of *GhCalS5* expression in cotton tissues.

### Overexpression of *GhCalS5* enhances cotton resistance to cotton aphids

**Figure 3:**
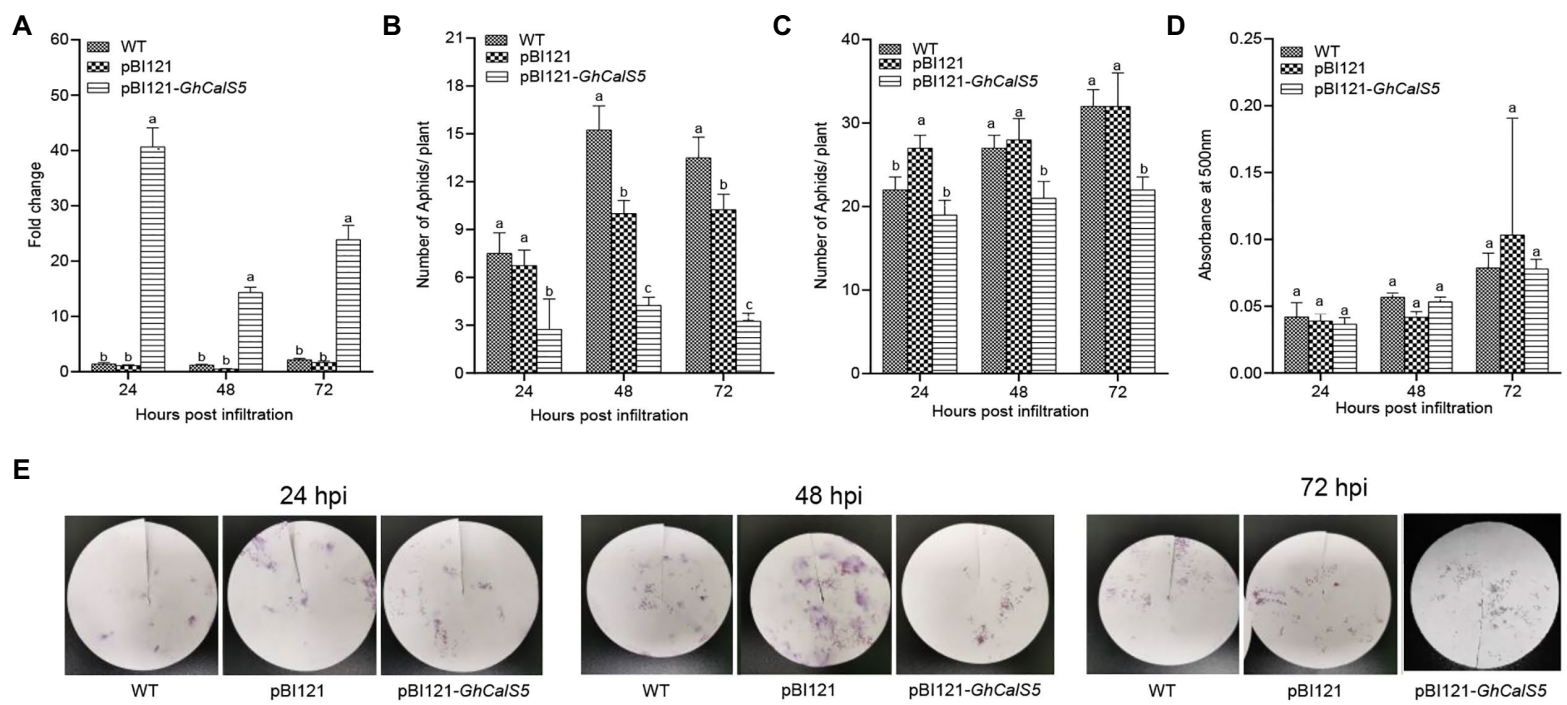
Overexpression GhCalS5 inhibited the population of cotton aphids in cotton plants. **(A)** transient expression of GhCalS5 in cotton leaves, **(B)** choice assay, **(C)** no-choice assay, **(D)** absorbance levels of aphid honeydew at 500 nm, **(E)** aphid honeydew deposition staining by ninhydrin. HPI, hours post infiltration. Number of plants used = 3 per treatment. One-way ANOVA analyzed data. Error bars represents the SD of three biological replicates. Different letters indicate significant differences (*p* < 0.05) based on Tukey’s HSD test.

To understand the involvement of *GhCalS5* in cotton resistance to aphids, *GhCalS5* was overexpressed using the transient expression method, in which *Agrobacterium* strain harboring pBI121 and pBI121-*GhCalS5* were infiltrated into cotton cotyledons. After 24, 48, and 72 h of infiltration, the transcript level of *GhCalS5* in cotton plants was significantly higher in *GhCalS5*-overexpressing plants than in control plants (pBI121) and wild-type, respectively (Figure 3A). To validate the role of *GhCalS5* in cotton response to aphid damage, aphid performance on wild-type, pBI121, and pBI121—*GhCalS5* plants was analyzed *via* host choice assay. In the no-choice assay, the population of aphids was significantly low in *GhCalS5*-overexpressing cotton plants at 48 and 72 h after infiltration compared to the wild-type and control plants, suggesting that the overexpression of *GhCalS5* in cotton plants reduced aphid offspring (Figure 3B). Additionally, the choice assay showed that aphids preferred feeding on the wild-type leaves and control leaves than on leaves from *GhCalS5*-overexpressing plants significantly at 48 and 72 h after post infiltration (Figure 3C). These results indicate that *GhCalS5* overexpression in cotton leaves improves cotton resistance against aphids. Then, we investigated the effects of *GhCalS5* gene overexpression on aphid honeydew excretion. Honeydew produced by aphids at 24, 48, and 72 h after feeding was quantified. However, no significant difference was detected in the honeydew absorbance in all plants after aphid feeding (Figures 3D,E).

### Silencing of *GhCalS5* increases cotton susceptibility to cotton aphids

**Figure 4:**
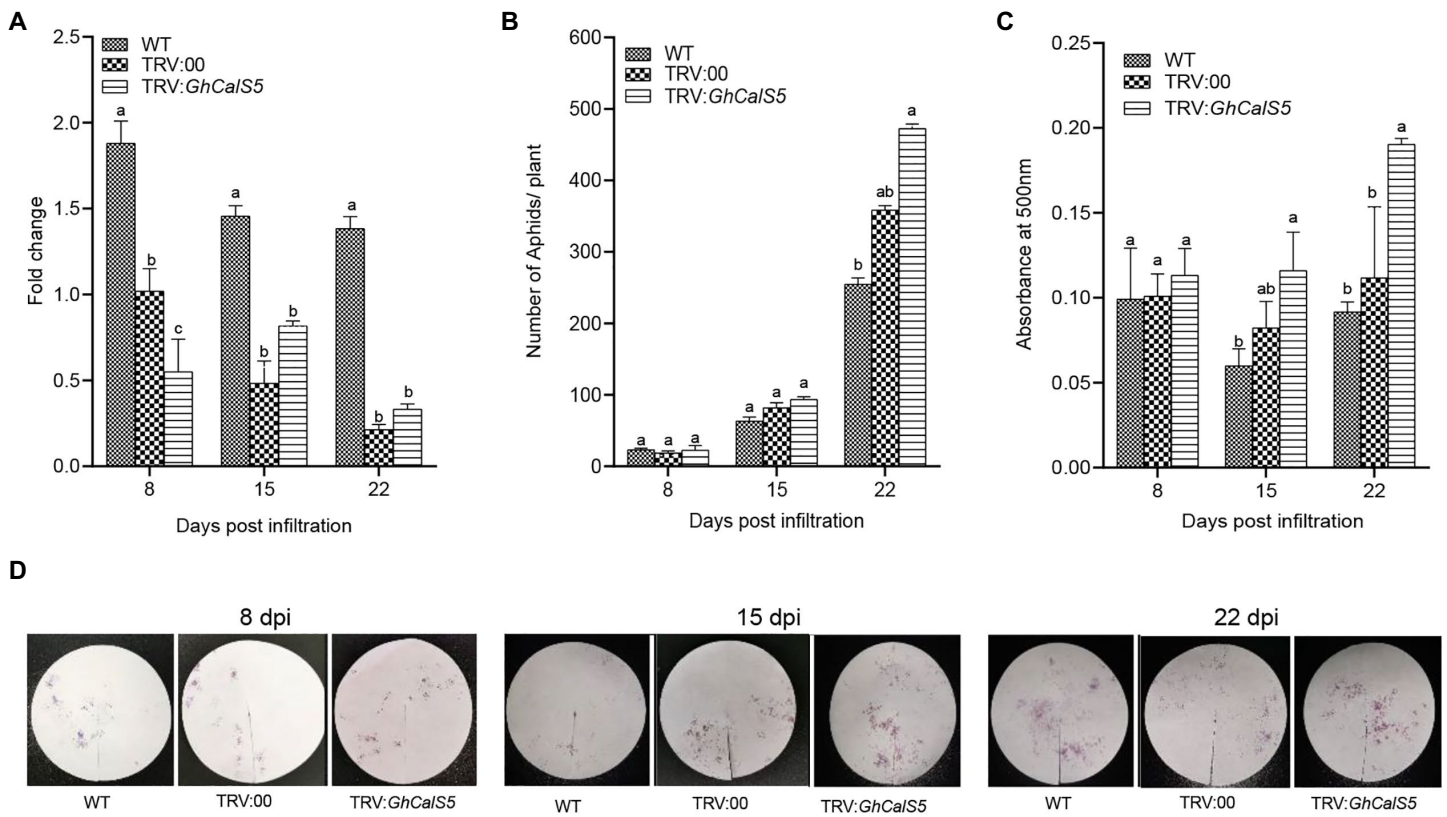
Silencing expression of GhCalS5 increased aphid population and feeding in cotton plants. **(A)** fold change in transcription of GhCalS5 in cotton leaves, **(B)** aphid no-choice assay, **(C)** absorbance levels of aphid honeydew at 500 nm **(D)** aphid honeydew deposition staining by ninhydrin. DPI, days post infiltration. Number of plants used = 3 per treatment. One-way ANOVA analyzed data. Error bars represents the SD of three biological replicates. Different letters indicate significant differences (*p* < 0.05) based on Tukey’s HSD test.

To further explore the role of *GhCalS5* in cotton response to aphids, the *GhCalS5* gene expression was silenced using the VIGS method. The transcriptional levels of *GhCalS5* in *GhCalS5*-silenced plants were significantly low compared to the wild-type and TRV: 00 plants at 8 and 15 days after injecting (Figure 4A). To further investigate the involvement of *GhCalS5* in plant response to cotton aphids, a no-choice assay test was performed on *GhCalS5*-silenced plants, control (TRV: 00), and wild-type plants. The population of aphids was higher in *GhCalS5*-silenced plants than in the TRV: 00 and wild-type plants. At 15 days after *A. tumefaciens* infiltration, the number of cotton aphids elevated rapidly on *GhCalS5*-silenced plants *and* reached an average of 473 aphids per plant at 22 d, while the number of cotton aphids on wild plants reached an average of 256 aphids per plant (Figure 4B). Additionally, we also determined the aphid feeding activity after silencing the *GhCalS5* gene in cotton plants. The honeydew absorbance was the same 8 days after *A. tumefaciens* infiltration and gradually increased. It was significantly higher in *GhCalS5*-silenced plants than wild-type cotton at 15 days and remained elevated up to 22 days after *A. tumefaciens* infiltration (Figures 4C,D). These results suggest that silencing *GhCalS5* enhances cotton susceptibility to aphids.

### *GhCalS5* is involved in callose formation in cotton response to cotton aphid damage

**Figure 5:**
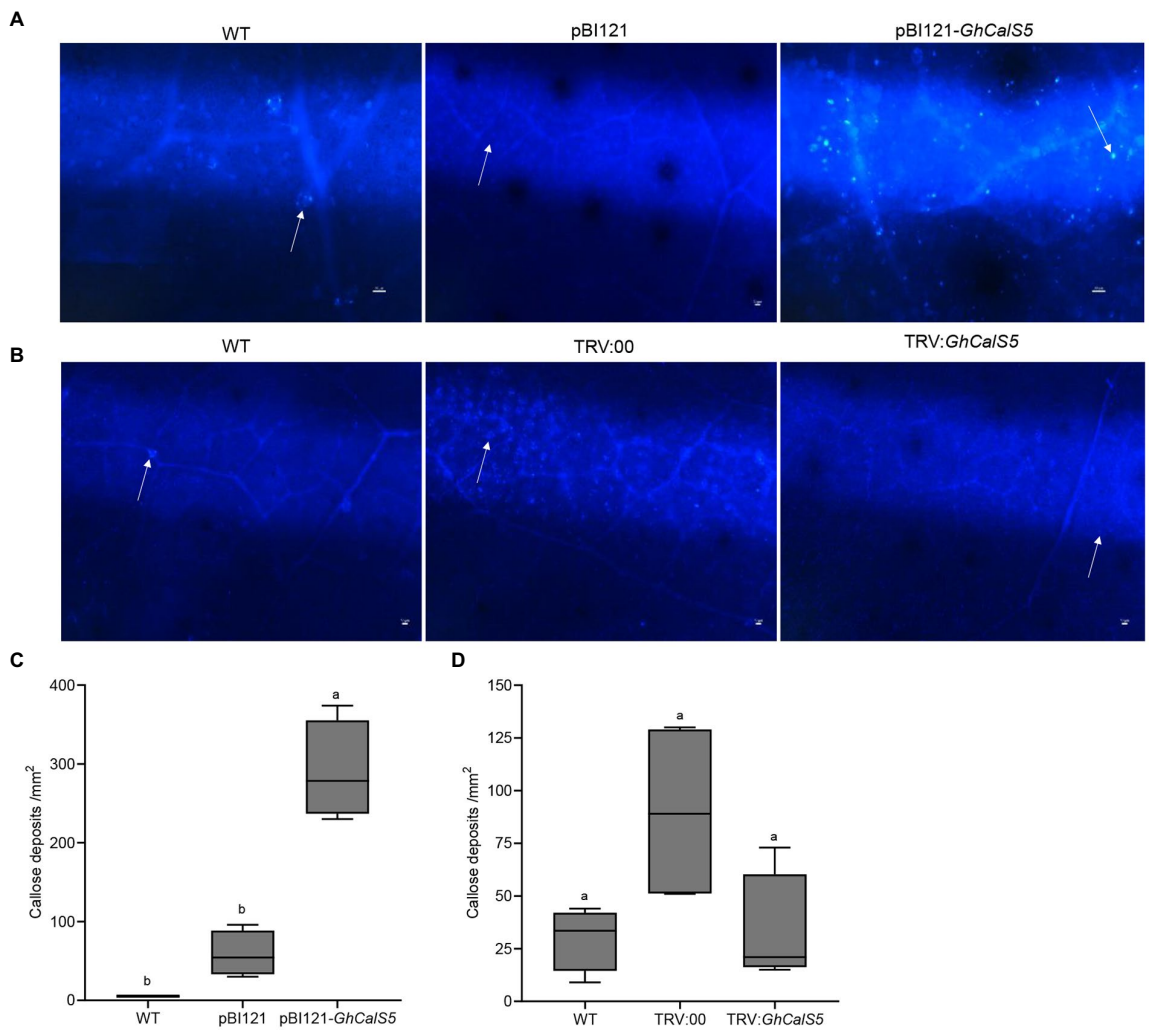
Callose deposition and quantification in *GhCalS5-*overexpressed or silenced cotton leaves. **(A)** Callose deposition in cotton leaves in WT, pBI121 (control), *GhCalS5*-overexpressed. **(B)** Callose deposition in cotton leaves in TRV: 00 and *GhCalS5*-silenced plants. **(B)** Callose deposits quantification in WT, pBI121 (control), and *GhCalS5*-overexpressed using ImageJ software. Callose deposits quantification in **(C)** WT, pBI121 (control), pBI121-GhCalS5 plants **(D)** WT, TRV: 00 (control), TRV:GhCalS5 plants using ImageJ software. One-way ANOVA analyzed data. Error bars represents the SD of three biological replicates. Different letters indicate significant differences (*p* < 0.05) based on Tukey’s HSD test. Values are shown per mm^2^ from the leaf of four independent plants. Scar bar: 50 μm.

Callose is considered an essential index of resistance to aphids, and its formation is closely related to callose synthase. To clarify callose deposition in *GhCalS5-*overexpressing plants, we visualized callose deposits in leaves through fluorescence microscopy. Callose deposits were higher on leaves from *GhCalS5*-overexpressing plants than in wild-type (WT) and control plants (pBI121; Figures 5A,B). These results correlated with *GhCalS5* transcript level after GV3101 infiltration and suggest that the overexpression of *GhCalS5* increases callose deposition in the leaves of cotton plants. Furthermore, we observed callose deposition in cotton leaves after silencing *GhCalS5* in cotton. Few callose deposits accumulated on leaves from *GhCalS5*-silenced plants, no significant difference in leaf disk callose deposits from wild-type plants. The results indicate that the silencing of *GhCalS5* in cotton reduces the number of callose deposits on cotton leaves (Figures 5A,C). In short, these results demonstrated that *GhCalS5* is involved in cotton in response to cotton aphid damage through callose formation.

## Discussion

Biotic and abiotic stresses in nature often challenge plants. Plants have gradually developed various defined and complex mechanisms to respond to these damages during a long period of coevolution (Erb and Reymond, 2019). Callose deposition is induced in plant tissue as a defense mechanism response to both biotic and abiotic stresses. Several studies have reported that callose deposition is induced by biotic and abiotic stress (Jacobs et al., 2003; Hao et al., 2008; Li et al., 2018; O’Lexy et al., 2018; Hunter et al., 2019). However, the function and mechanism of callose formation in the plant defense response to insect pests have still not been studied in depth (Liu et al., 2017). Therefore, we performed a study on the role of callose synthase gene *GhCalS5* in cotton response to aphid infestation.

Callose has been reported to play a role in plant defense response against insect pests. For instance, the Russian wheat aphid’s (*Diuraphis noxia*) sustained phloem feeding could reduce transport in *Triticum aestivum* by influencing callose formation. However, the mechanism of callose formation is not described (Botha and Matsiliza, 2004). The *Arabidopsis* transcriptome analysis in response to the nymphs of *Bemisia tabaci type B* showed that callose synthase gene RNAs accumulated and callose deposition was observed in SLWF-infested tissue, suggesting that callose synthase is involved in callose formation in *A. thaliana* (Kempema et al., 2007). In addition, exogenous ABA suppressed β-1,3-glucanase and induced synthase activity, and promoted callose deposition (Liu et al., 2017). Upon aphid attack in the pepper plant, callose synthase genes were induced in the leaves (Sun et al., 2018). However, there are few reports on the function of callose synthase genes in cotton insect resistance that have been made. In this study, we cloned a cotton callose synthase gene *GhCalS5* and demonstrated that the *GhCalS5* gene was upregulated in cotton plants at 48 h after cotton aphid infestation (Figures 2B,C). The results confirmed that *GhCalS5* is involved in cotton response to cotton aphid attack. Moreover, the *GhCalS5* transcript was upregulated significantly at 24 h after SA treatment (Figure 2D), suggesting that the *GhCalS5* transcript is related to SA, which was consistent with the results that direct exogenous application of SA improve plasmodesmata closure and callose deposition (Wang et al., 2013).

Upon aphid infestation, plant defense response is often reflected by reduced non-preference (antixenosis) in a choice test and by reduced offspring production (antibiosis) in a no-choice test (Lei et al., 2014). In this study, when aphids were given a choice of host plants (Figure 3B), approximately twice as many cotton aphids preferred to feed on wild-type than *GhCalS5* overexpressed plants. Secondly, aphids are likely to have low fecundity and die early due to difficulties taking up nutrients when feeding on plants containing phloem-based resistance (Sun et al., 2018). This is in line with our observations that the overexpression of *GhCalS5* resulted in an impaired aphid population after 72 h post infiltration in the no-choice tests assay (Figure 3C). To further validate the role of *GhCalS5* in cotton response to aphid attack, *GhCalS5*-silenced plants were exposed to aphid feeding. The population of aphid feeding on *GhCalS5-*silenced plants was higher than the aphids feeding on wild-type plants, and aphid feeding on *GhCalS5*-silenced plants excreted more honeydew than aphid feeding on wild-type, which indicates that *GhCalS5* silence contributed to feeding of cotton aphids (Figure 4).

Callose plays an important role in aphid resistance in plants. Previous studies have shown that brown planthopper’s (BPH) feeding on rice resistant cultivars was interrupted due to callose deposition in the sieve plates, BPH preferred to feed on the susceptible variety of plants (Hao et al., 2008). We conclude that the preference of aphids to feed on wild-type plants and the reduced population were due to callose deposition in the leaves in *GhCalS5*-overexpressing plants (Figure 3). To confirm this hypothesis, callose deposits were stained in cotton leaves. Callose deposition in *GhCalS5* overexpressed plants was significantly more than that in WT plants (Figures 5A,B), suggesting that the overexpression of *GhCalS5* could promote callose deposition. Depleting DIMBOA-Glc reserves and inducing HDMBA-Glc production by caterpillar feeding in maize induces aphid susceptibility by suppressing callose (Meihls et al., 2013). Aphids grew better on maize cultivars with low DIMBOA-Glc and high HDMBOA-Glc levels, which coincides with low callose inducibility in the genotypes (Maag et al., 2016). In this study, we found a slight difference in callose deposits between the *GhCalS5*-silenced and WT plants (Figures 5A,C). The low callose deposition level in these plants may have resulted in an increased population of aphid feeding on *GhCalS5*-silenced plants. Of course, further research is needed.

In summary, *GhCalS5* could be involved in controlling aphids in cotton plants. However, there is still a research gap on the detailed mechanisms through which callose acts during defenses. Therefore, there is a need to unceasingly study the callose synthase genes to understand their function in plant-herbivore interaction better.

## Data availability statement

The raw data supporting the conclusions of this article will be made available by the authors, without undue reservation.

## Author contributions

JZ and YY designed the research. NITM, ZH, and HZ performed the experiments with the assistance of YZ, XL, YuW, YiW, TL, and JL. JZ, NITM, and XW analyzed the data and wrote the manuscript. All authors contributed to the article and approved the submitted version.

## Funding

This work was supported by grants from the National Natural Science Foundation of China (grant no. 31471783) and the Foundation of Guangxi Key Laboratory of Biology for Crop Diseases and Insect Pests (grant no. 20–065-30-KF-01).

## Conflict of interest

The authors declare that the research was conducted in the absence of any commercial or financial relationships that could be construed as a potential conflict of interest.

## Publisher’s note

All claims expressed in this article are solely those of the authors and do not necessarily represent those of their affiliated organizations, or those of the publisher, the editors and the reviewers. Any product that may be evaluated in this article, or claim that may be made by its manufacturer, is not guaranteed or endorsed by the publisher.

## Footnotes

^1^ https://blast.ncbi.nlm.nih.gov/Blast.cgi

## Supplementary materials

The Supplementary materials for this article can be found online at: https://www.frontiersin.org/articles/10.3389/fpls.2022.892630/full#supplementary-material
