# Supplementary material for: GhCalS5 is involved in cotton response to aphid attack through mediating callose formation
Source: Front Plant Sci. 2022 Jul 20;13:892630. doi: 10.3389/fpls.2022.892630 (PMC9350506; doi:10.3389/fpls.2022.892630)
Supplement: Supplementary file 2 [file Data_Sheet_2.PDF]

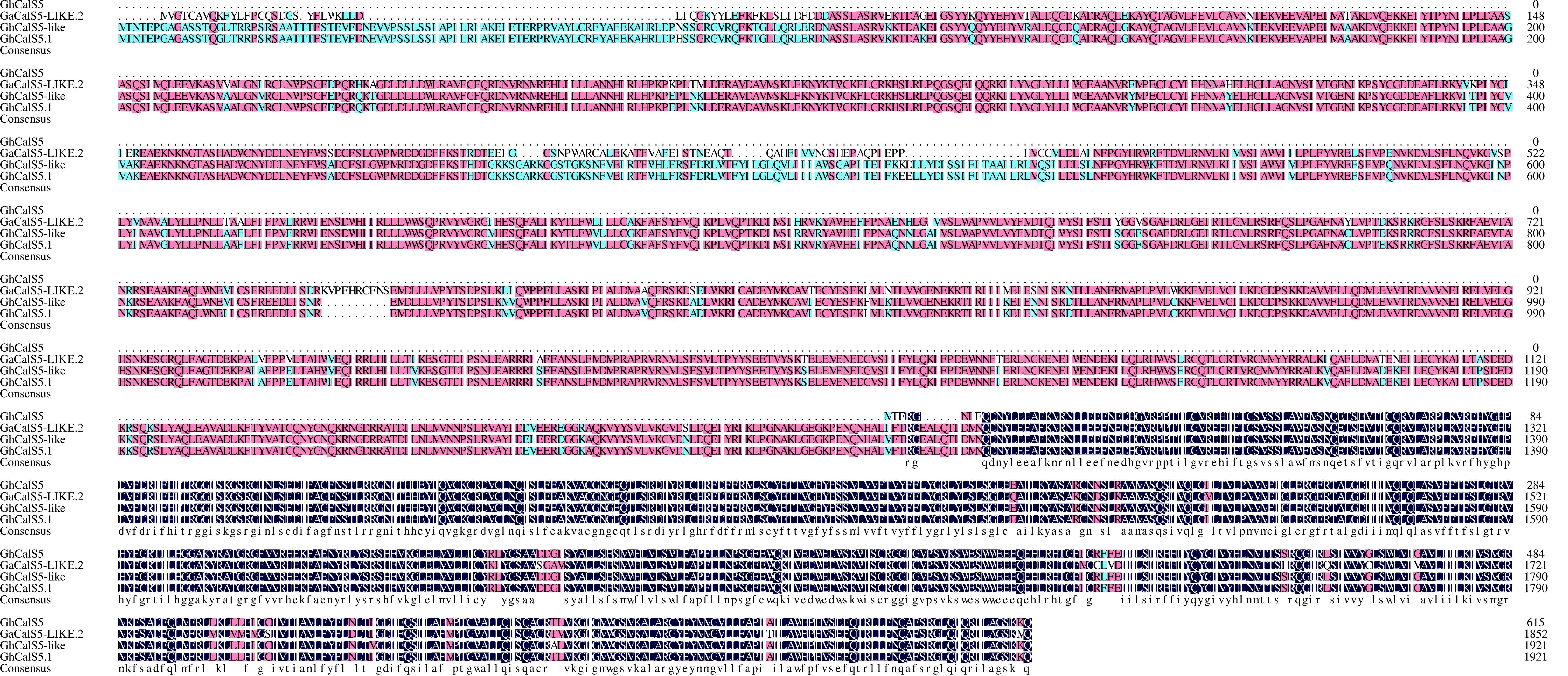
GhCalS5 0
GaCalS5-LIKE.2 148
GhCalS5-like 200
GhCalS5.1 200
Consensus
GhCalS5 0
GaCalS5-LIKE.2 348
GhCalS5-like 400
GhCalS5.1 400
Consensus
GhCalS5 0
GaCalS5-LIKE.2 522
GhCalS5-like 600
GhCalS5.1 600
Consensus
GhCalS5 0
GaCalS5-LIKE.2 721
GhCalS5-like 800
GhCalS5.1 800
Consensus
GhCalS5 0
GaCalS5-LIKE.2 921
GhCalS5-like 990
GhCalS5.1 990
Consensus
GhCalS5 0
GaCalS5-LIKE.2 1121
GhCalS5-like 1190
GhCalS5.1 1190
Consensus
GhCalS5 84
GaCalS5-LIKE.2 1321
GhCalS5-like 1390
GhCalS5.1 1390
Consensus
GhCalS5 284
GaCalS5-LIKE.2 1521
GhCalS5-like 1590
GhCalS5.1 1590
Consensus
GhCalS5 484
GaCalS5-LIKE.2 1721
GhCalS5-like 1790
GhCalS5.1 1790
Consensus
GhCalS5 615
GaCalS5-LIKE.2 1852
GhCalS5-like 1921
GhCalS5.1 1921
Consensus
