# Supplementary material for: GhCalS5 is involved in cotton response to aphid attack through mediating callose formation
Source: Front Plant Sci. 2022 Jul 20;13:892630. doi: 10.3389/fpls.2022.892630 (PMC9350506; doi:10.3389/fpls.2022.892630)
Supplement: Supplementary file 3 [file Data_Sheet_3.PDF]

Alignment of GhCalS5(upper line) and GaCalS5-LIKE.2(lower line)
Identity=93.99%(579/616) Similar residues=5.03%(31/616)
Gap=66.76%(1237/1853)
----

1 ....
..

1
MVGTCAVQKFYLFPCQSDGSYFLWKLLDLIQGKYYLEFKFKLSLIDFDDDASSLASRVEK

1 ....
..

61
TDAGEIGSYYKQYYEHYVTALDQGDKADRAQLEKAYQTAGVLFEVLCAVNNTEKVEEVAP

1 ....
..

121
EIMATAKDVQEKKEIYTPYNILPLDAASASQSIMQLEEVKASVVALGNIRGLNWPSGFDP

1 ....
..

181
QRHKAGDLDLLDWLRAMFGFQRDNVRNMREHLILLLANNHIRLHPKPKPLTMLDERAVDA

1 ....
..

241
VMSKLFKNYKTWCKFLGRKHSLRLPQGSQEIQQRKILYMGLYLLIWGEAANVRFMPECLC

1 ....
..

301
YIFHNMAHELHGLLAGNVSIVTGENIKPSYGGDDEAFLRKVVKPIYCIIEREAEKNKNGT

1 ....
..

361
ASHADWCNYDDLNEYFWSSDCFSLGWPMRDDGDFFKSTRDTEEIGCSNPWARCALEKATF

1 ....
..

421
VAFEISTNEAQTQAHFIVVNCSHEPAQPIEPPHVGCVLDLAINFPGYHRWRFTDVLRNVL

1 ....
..

481
KIVVSIAWVIILPLFYVRELSFVPENVKDMLSFLNQVKGVSPLYVMAVALYLLPNLLTAA

1 ....
..

541
LFIFPMLRRWIENSDWHIIRLLLWWSQPRVYVGRGIHESQFALIKYTLFWLILLCAKFAF

1 ....
..

601
SYFVQIKPLVQPTKDIMSIHRVKYAWHEFFPNAENHLGVVSLWAPVVLVYFMDTQIWYSI

1 ....
..

661
FSTIYGGVSGAFDRLGEIRTLGMLRSRFQSLPGAFNAYLVPTDKSRKRGFSLSKRFAEVT

1 ....
..

721
ANRRSEAAKFAQLWNEVICSFREEDLISDRKVPFHRCFNSEMDLLLVPYTSDPSLKLIQW

1 ....
..

781
PPFLLASKIPIALDMAAQFRSKDSELWKRICADEYMKCAVTECYESFKLVLNTLVVGENE

1 ....
..

841
KRTIRIIIMEIESNISKNTLLANFRMAPLPVLWKKFVELVGILKDGDPSKKDAVVFLLQD

1 ....
..

901
MLEVVTRDMMVNEIRELVELGHSNKESGRQLFAGTDEKPALVFPPVLTAHWVEQIRRLHI

1 ....
..

961
LLTIKESGTDIPSNLEARRRIAFFANSLFMDMPRAPRVRNMLSFSVLTPYYSEETVYSKT

1 ....
..

1021
ELEMENEDGVSIIFYLQKIFPDEWNNFTERLNCKENEIWENDEKILQLRHWVSLRGQTLC

1 ....
..

1081
RTVRGMMYYRRALKIQAFLDMATENEILEGYKAILTASDEDKRSQKSLYAQLEAVADLKF

1 ....
..

1141
TYVATCQNYGNQKRNGDRRATDILNLMVNNPSLRVAYIDDVEEREGGRAQKVYYSVLVKG

1 ....MTFRG.......NIFQDNYLEEAFKMR
NLL

:.| :: |||||||||||||||

1201
VDSLDQEIYRIKLPGNAKLGEGKPENQNHALIFTRGEALQTIDMNQDNYLEEAFKMRNLL

24
EEFNEDHGVRPPTILGVREHIFTGSVSSLAWFMSNQETSFVTIGQRVLARPLKVRFHYGH

||||||||||||||||||||||||||||||||||||||||||||||||||||||||||||
1261
EEFNEDHGVRPPTILGVREHIFTGSVSSLAWFMSNQETSFVTIGQRVLARPLKVRFHYGH

84
PDVFDRIFHITRGGISKGSRGINLSEDIFAGFNSTLRRGNITHHEYIQVGKGRDVGLNQI

||||||||||||||||||||||||||||||||||||||||||||||||||||||||||||
1321
PDVFDRIFHITRGGISKGSRGINLSEDIFAGFNSTLRRGNITHHEYIQVGKGRDVGLNQI

144
SLFEAKVACGNGEQTLSRDIYRLGHRFDFFRMLSCYFTTVGFYFSSMLVVFTVYFFLYGR

||||||||||||||||||||||||||||||||||||||||||||||||||||||||||||
1381
SLFEAKVACGNGEQTLSRDIYRLGHRFDFFRMLSCYFTTVGFYFSSMLVVFTVYFFLYGR

204
LYLSLSGLEEAILKYASARGNNSLRAAMASQSIVQLGILTVLPMVMEIGLERGFRTALGD

|||||||||:||||||||:||:||:||||||||||||:||||||||||||||||||||||
1441
LYLSLSGLEQAILKYASAKGNDSLKAAMASQSIVQLGVLTVLPMVMEIGLERGFRTALGD

264
IIIMQLQLASVFFTFSLGTRVHYFGRTILHGGAKYRATGRGFVVRHEKFAENYRLYSRSH

||||||||||||||||||||||||||||||||||||||||||||||||||||||||||||
1501
IIIMQLQLASVFFTFSLGTRVHYFGRTILHGGAKYRATGRGFVVRHEKFAENYRLYSRSH

324
FVKGLELMVLLICYRLYGSAADDGISYALLSFSMWFLVLSWLFAPFLLNPSGFEWQKIVE

||||||||||||||::|||||.:::|||||||||||||||||||||||||||||||||||
1561
FVKGLELMVLLICYKIYGSAASGAVSYALLSFSMWFLVLSWLFAPFLLNPSGFEWQKIVE

384
DWEDWSKWISCRGGIGVPSVKSWESWWEEEQEHLRHTGFIGRFFEIILSIRFFIYQYGIV

|||||||||||||||||||||||||||||||||||||||:| :.:|||||||||||||||
1621
DWEDWSKWISCRGGIGVPSVKSWESWWEEEQEHLRHTGFMGCLVDIILSIRFFIYQYGIV

444
YHLNMTTSSRQGIRLSIVVYGLSWLVIGAVLIILKIVSMGRMKFSADFQLMFRLLKLLLF
     ||||||||| |||||
|||||.|||||||.||||||||||||||||||||||||||:||::|
1681
YHLNMTTSIRQGIRQSIVVYCLSWLVIVAVLIILKIVSMGRMKFSADFQLMFRLVKLVMF

504
IGCIVTIAMLFYFLNLTIGDIFQSILAFMPTGWALLQISQACRTLVKGIGMWGSVKALAR
     :|.|||||||||||:|||||||||||||:|||||||||||||||:||||||||||||
|||
1741
VGSIVTIAMLFYFLDLTIGDIFQSILAFVPTGWALLQISQACRTVVKGIGMWGSVKALAR

564   GYEYMMGVLLFAPIAILAWFPFVSEFQTRLLFNQAFSRGLQIQRILAGSKKQA
      |||||||||||||.||||||||||||||||||||||||||||||||||||.||

1801 GYEYMMGVLLFAPITILAWFPFVSEFQTRLLFNQAFSRGLQIQRILAGSKMQA
