# Supplementary material for: GhCalS5 is involved in cotton response to aphid attack through mediating callose formation
Source: Front Plant Sci. 2022 Jul 20;13:892630. doi: 10.3389/fpls.2022.892630 (PMC9350506; doi:10.3389/fpls.2022.892630)
Supplement: Supplementary file 4 [file Data_Sheet_4.PDF]

Alignment of GhCalS5(upper line) and GhCalS5.1(lower line)
Identity=98.86%(609/616) Similar residues=0.65%(4/616)
Gap=67.95%(1306/1922)
----

1   ....
..

1
MTNTEPGAGASSTQGLTRRPSRSAATTTFSTEVFDNEVVPSSLSSIAPILRIAKEIETER

1   ....
..

61
PRVAYLCRFYAFEKAHRLDPHSSGRGVRQFKTGLLQRLERDNASSLASRVKKTDAKEIGS

1   ....
..

121
YYQQYYEHYVRALDQGDQADRAQLGKAYQTAGVLFEVLCAVNKTEKVEEVAPEIMAAAKD

1   ....
..

181
VQEKKEIYTPYNILPLDAAGASQSIMQLEEVKASVAALGNVRGLNWPSGFEPQRQKTGDL

1   ....
..

241
DLLDWLRAMFGFQRDNVRNMREHLILLLANNHIRLHPKPEPLNKLDERAVDAVMSKLFKN

1   ....
..

301
YKTWCKFLGRKHSLRLPQGSQEIQQRKILYMGLYLLIWGEAANVRYMPECLCYIFHNMAY

1   ....
..

361
ELHGLLAGNVSIVTGENIKPSYGGDDEAFLRKVITPIYCVVAKEAEKNKNGTASHADWCN

1   ....
..

421
YDDLNEYFWSADCFSLGWPMRDDGDFFKSTHDTGKKSGARKCGSTGKSNFVEIRTFWHLF

1 ....
..

481
RSFDRLWTFYILGLQVLIIIAWSGAPITEIFKEELLYDISSIFITAAILRLVQSILDLSL

1 ....
..

541
NFPGYHRWKFTDVLRNVLKIIVSIAWVIVLPLFYVREFSFVPQNVKDMLSFLNQVKGINP

1 ....
..

601
LYIMAVGLYLLPNLLAAFLFIFPMFRRWIENSDWHIIRLLLWWSQPRVYVGRGMHESQFA

1 ....
..

661
LIKYTLFWVLLLCGKFAFSYFVQIKPLVQPTKDIMSIRRVRYAWHEIFPNAQNNLGAIVS

1 ....
..

721
LWAPVVLVYFMDTQIWYSIFSTISGGFSGAFDRLGEIRTLGMLRSRFQSLPGAFNACLVP

1 ....
..

781
TEKSRRRGFSLSKRFAEVTANKRSEAAKFAQLWNEIICSFREEDLISNREMDLLLVPYTS

1 ....
..

841
DPSLKMVQWPPFLLASKIPIALDMAVQFRSKDADLWKRICADEYMKCAVIECYESFKIVL

1 ....
..

901
KTLVVGENEKRTIRIIIKEIENNISKDTLLANFRMAPLPVLCKKFVELVGILKDGDPSKK

1   ....
..

961
DAVVFLLQDMLEVVTRDMMVNEIRELVELGHSNKESGRQLFAGTDEKPAIAFPPELTAHW

1   ....
..

1021
IEQIRRLHILLTVKESGTDIPSNLEARRRISFFANSLFMDMPRAPRVRNMLSFSVLTPYY

1   ....
..

1081
SEETVYSKSELEMENEDGVSIIFYLQKIFPDEWNNFIERLNCKENEIWENDEKILQLRHW

1   ....
..

1141
VSFRGQTLCRTVRGMMYYRRALKVQAFLDMADEKEILEGYKAILTPSDEDKKSQRSLYAQ

1   ....
..

1201
LEAVADLKFTYVATCQNYGNQKRNGDRRATDILNLMVNNPSLRVAYIDEVEERDGGKAQK

1   ....MTFRG.......NIFQDN
YLE

:.| :: ||||||
1261
VYYSVLVKGVDNLDQEIYRIKLPGNAKLGEGKPENQNHALVFTRGEALQTIDMNQDNYLE

15
EAFKMRNLLEEFNEDHGVRPPTILGVREHIFTGSVSSLAWFMSNQETSFVTIGQRVLARP

||||||||||||||||||||||||||||||||||||||||||||||||||||||||||||
1321
EAFKMRNLLEEFNEDHGVRPPTILGVREHIFTGSVSSLAWFMSNQETSFVTIGQRVLARP

75
LKVRFHYGHPDVFDRIFHITRGGISKGSRGINLSEDIFAGFNSTLRRGNITHHEYIQVGK

||||||||||||||||||||||||||||||||||||||||||||||||||||||||||||
1381
LKVRFHYGHPDVFDRIFHITRGGISKGSRGINLSEDIFAGFNSTLRRGNITHHEYIQVGK

135
GRDVGLNQISLFEAKVACGNGEQTLSRDIYRLGHRFDFFRMLSCYFTTVGFYFSSMLVVF

||||||||||||||||||||||||||||||||||||||||||||||||||||||||||||
1441
GRDVGLNQISLFEAKVACGNGEQTLSRDIYRLGHRFDFFRMLSCYFTTVGFYFSSMLVVF

195
TVYFFLYGRLYLSLSGLEEAILKYASARGNNSLRAAMASQSIVQLGILTVLPMVMEIGLE

||||||||||||||||||||||||||||||||||||||||||||||||||||||||||||
1501
TVYFFLYGRLYLSLSGLEEAILKYASARGNNSLRAAMASQSIVQLGILTVLPMVMEIGLE

255
RGFRTALGDIIIMQLQLASVFFTFSLGTRVHYFGRTILHGGAKYRATGRGFVVRHEKFAE

||||||||||||||||||||||||||||||||||||||||||||||||||||||||||||
1561
RGFRTALGDIIIMQLQLASVFFTFSLGTRVHYFGRTILHGGAKYRATGRGFVVRHEKFAE

315
NYRLYSRSHFVKGLELMVLLICYRLYGSAADDGISYALLSFSMWFLVLSWLFAPFLLNPS

||||||||||||||||||||||||||||||||||||||||||||||||||||||||||||
1621
NYRLYSRSHFVKGLELMVLLICYRLYGSAADDGISYALLSFSMWFLVLSWLFAPFLLNPS

375
GFEWQKIVEDWEDWSKWISCRGGIGVPSVKSWESWWEEEQEHLRHTGFIGRFFEIILSIR

||||||||||||||||||||||||||||||||||||||||||||||||||||||||||||
1681
GFEWQKIVEDWEDWSKWISCRGGIGVPSVKSWESWWEEEQEHLRHTGFIGRFFEIILSIR

435
FFIYQYGIVYHLNMTTSSRQGIRLSIVVYGLSWLVIGAVLIIILKIVSMGRMKFSADFQLM

||||||||||||||||||||||||||||||||||||||||||||||||||||||||||||
1741
FFIYQYGIVYHLNMTTSSRQGIRLSIVVYGLSWLVIGAVLIIILKIVSMGRMKFSADFQLM

495
FRLLKLLLFIGCIVTIAMLFYFLNLTIGDIFQSILAFMPTGWALLQISQACRTLVKGIGM

```
||||||||||||||||||||||||||||||||||||||||||||||||||||||||||||
1801
FRLLKLLLFIGCIVTIAMLFYFLNLTIGDIFQSILAFMPTGWALLQISQACRTLVKGIGM

555
WGSVKALARGYEYMMGVLLFAPIAILAWFPFVSEFQTRLLFNQAFSRGLQIQRILAGSKK

||||||||||||||||||||||||||||||||||||||||||||||||||||||||||||
1861
WGSVKALARGYEYMMGVLLFAPIAILAWFPFVSEFQTRLLFNQAFSRGLQIQRILAGSKK

615   QA
      ||
1921  QA
```
