# Supplementary material for: GhCalS5 is involved in cotton response to aphid attack through mediating callose formation
Source: Front Plant Sci. 2022 Jul 20;13:892630. doi: 10.3389/fpls.2022.892630 (PMC9350506; doi:10.3389/fpls.2022.892630)
Supplement: Supplementary file 6 [file Data_Sheet_6.PDF]

Alignment of GhCalS5(upper line) and GaCalS5-Like2(lower line)
Identity=64.37%(1185/1841) Gap=86.86%(12173/14014)
----

1   ....
..

1
ATGGTTGGTACTTGGTACTTGGTACTTGGTTAGAGAATTGAATCCGCCAAGACAACAGAG

1   ....
..

61
AGGGAATTTTTTTGCCTAGTAATTACTAGTAATCTATGCCAAAGGCTTTTGTCACAGTGT

1   ....
..

121
CAATCAGATGTTTAGAAGACACCACAACTTGAAAATTACGACCAAAAAGGGACTGCATTT

1   ....
..

181
AAATTAAATTCCATACTAATTGTTCCTCTTACCAGTGCGGTACAGAAATTCTACTTGTTC

1   ....
..

241
CCGTGCCAGAGTGATGGGTCTTATTTTCTATGGAAACTATTGGATCTCATTCAAGGAAAA

1   ....
..

301
TATTACTTGGAGTTCAAGTTTAAGTTATCACTGATTGATTTTGATGTATGTTTGCATGCA

1   ....
..

361
TACATCTATTTTCATCTTGTTTGTATGTATATGCTTATTTTGCACTTTATAAAAGCCATA

1   ....
..

421
TACAATTTGGGCTTTTGGTCCTTTTGGATATTCAATTGCTTTTAGCTAAGGTTTCTGTTG

1 ....
..

481
CCCTGTTTATCTGTTTCTTCTATTCTTTGGTATGAGAAGATAACAACAATTAAACAAGGT

1 ....
..

541
TTGGTTGGTCTCCCTTCCATGTTTTATTTCATTCCAAGTATATCAAAATTTTTGCTACCA

1 ....
..

601
TCATTCACAATCTCACAACTATAAGCATAACTTGATGGCACAAACATTTTTAGCATTTCA

1 ....
..

661
CACCATGATTAATATTTTGGACTGTAGGATGATGCATCCAGTCTTGCCTCTCGAGTAGAA

1 ....
..

721
AAGACTGATGCAGGGGAAATTGGGAGCTACTATAAACAGTATTATGAACACTATGTTACA

1 ....
..

781
GCACTGGACCAGGGAGATAAGGCAGACAGGTAACTAATATCACAAGGGGTTTCCACTACA

1 ....
..

841
CTTGAGAATGTCAACTAATTTCCTTTCCCGATTTGTTGACAGAGCTCAACTTGAGAAAGC

1 ....
..

901
TTACCAAACAGCTGGAGTGCTTTTTGAAGTTCTTTGTGCCGTTAATAACACTGAGAAAGT

1 ....
..

961
TGAAGAAGTTGCTCCCGAGGTTAGTCCTGTGTTTAGTAATTCCATCAAGTTTTTATTTAC

1 ....
..

1021
AATGAATAGCTGATTATAATATTGCCAATTTTGATGGCATAAAATGTAATTTCAAATGAC

1 ....
..

1081
ATTTCCTCGGGTAGATCATGGCAACTGCCAAAGATGTTCAAGAAAAGAAGGAAATCTACA

1 ....
..

1141
CTCCCTACAACATTCTTCCTTTGGATGCTGCCAGTGCTTCACAGTCTATCATGCAGCTTG

1 ....
..

1201
AGGAGGTAACAATTCATAGTATCATGTGTCATTCTGCTGATTAAGTGGCGGATCCAAAAA

1 ....
..

1261
AAAAAATTGTCTGTGAGATCATATTTAATTAGCATGTGGAAAAGATATGCATACCTTAAA

1 ....
..

1321
TTATGAAGAAATTGACATAAAATTTCAGACACTTTGAACTAGCTCTACTCTGCTCTGCTG

1 ....
..

1381
CTGTAATGACTCAGTGTCATTCCAGCATCTTATTTGTTGGCGTATTGTTTTAGATGCTAC

1 ....
..

1441
TTGAACTTTACTTTAATATACAGGTTAAGGCTTCTGTGGTTGCACTAGGGAACATTCGTG

1 ....
..

1501
GTTTGAACTGGCCTTCGGGATTTGATCCACAAAGGCATAAGGCTGGAGACTTGGACCTTT

1 ....
..

1561
TGGATTGGTTAAGAGCCATGTTTGGATTCCAGGTAAGTTTTTAACATCCCCATGCCCACG

1 ....
..

1621
AGCCAGAAAAAGGCTTGTTGTTGAATGAATAAATGGTCTTCTTCAAATATCATGCATTTT

1 ....
..

1681
GGCTTTCATCACTGCAGAGAGACAATGTCAGGAACATGAGGGAGCACTTGATTTTGCTGC

1 ....
..

1741
TTGCCAATAATCATATAAGACTCCACCCCAAGCCCAAACCTCTTACTATGGTATGCACAG

1 ....
..

1801
GTGTTTAATCTCTTTTTCGCGTGCCATTGGTCACTGAAGTTTCAAATGGAAGCCTAGATA

1 ....
..

1861
TAGTTTCACTTGTTCTTCTAAGCTTAAGCTTGTATACTGATTAAACTTATTTCTTTTATG

1 ....
..

1921
TAGCTTGATGAACGAGCAGTTGATGCTGTTATGAGCAAGCTTTTTAAGAATTACAAAACA

1 ....
..

1981
TGGTGTAAATTCTTAGGACGTAAACATAGTTTAAGGTAAAAAATAACAACTTATACATCT

1 ....
..

2041
AATTACTGGTGGATATTTCCGTACATTACTATTGTGCTCTTCAACTCTACTTTCTGTTAA

1 ....
..

2101
ATCGAAACAATTTGTGGTCTAATTTGTACTGACTCATTCATGTATAGGCTGCCTCAAGGC

1 ....
..

2161
TCTCAAGAAATACAACAGAGGAAGATACTATATATGGGATTATATCTTCTCATCTGGGGT

1 ....
..

2221
GAAGCAGCTAATGTCCGGTTCATGCCAGAATGTCTATGCTACATTTTTCATAATGTAAGT

1 ....
..

2281
ATTCTTCGAATATCTAATGCTACCCATTTTCTTCTGTTCGACAAGGTTCTGCTTTTGTTC

1 ....
..

2341
ACCTATTATTGTTTCATTTCAGCAAGAAATATGTAGTTTGTAGTATTTTGAATTACATCC

1 ....
..

2401
CATGATACTCTCAGATGGCACATGAACTCCATGGCCTGTTGGCTGGAAATGTTAGCATAG

1 ....
..

2461
TGACTGGAGAAAATATCAAGCCCTCATATGGTGGAGATGATGAGGCTTTCTTGAGGAAGG

1 ....
..

2521
TTGTGAAACCGATTTACTGTATCATTGAAAGGGTATGCTTGATGACGAATTTTCTTGGAC

1 ....
..

2581
ATTGGATATTGTGTTCTAGATACAAATAAGGGAGTTCAAATATTGCAGGAAGCCGAAAAG

1 ....
..

2641
AACAAAAACGGAACGGCTTCTCATGCAGACTGGTGTAATTATGATGATCTAAACGAGTAT

1 ....
..

2701
TTCTGGTTTGTATGGTTTTAATATAAAAATTTGATTGGTTTAAGGGTATCAATTTTAGTT

1 ....
..

2761
CTAATTGAGTTATGATGAAATTCTTTTCAGGTCTTCTGATTGCTTCTCTCTTGGATGGCC

1 ....
..

2821
TATGCGTGATGATGGTGACTTTTTCAAATCAACACGTGACACGGTAGTCCTTAGTGGTTG

1 ....
..

2881
TCAGCACTTTTCCTAAATAAGCTAGTGATTGTGTTGCTTAATGCAGCTACTATGCCAGGG

1 ....
..

2941
AAAGAAGACTTCTCGAAGAAAATGTAGAAGCACCGGCAAATCAAATTTTGTTGAGATTAG

1 ....
..

3001
AACATTTTGGCACCTCTTTCGAAGTTTTGATCGACTATGGACTTTTTATATTCTAGGTTT

1 ....
..

3061
GCAGGTATTAAAATGTGATCTTTGCTCTTCGTTTTTGATTCTTGTACATATTAATATTCC

1 ....
..

3121
AATATTTTATGTTTAGGTAATGATCATTATTGCATGGAGTGGAGCTTCGTTAACAGAAAT

1 ....
..

3181
CTTTCAAAAGGATTTATTGTATGATATATCCAGTATTTTCATCACAGCGGCCATTTTGCG

1 ....
..

3241
TTTCATTCAGAGTATGATATTCTTACTTAAATTGCTGTCTTCTACGAAGTTTATTTTTCC

1 ....
..

3301
TCCAGAATTTTATTTGCTTTGTCTCTTTGGCTTCTTAATAGCCAATTATTCTCTGAAATT

1 ....
..

3361
TGCTAAGACTGGAATATTAATATTCAAGAGGTTGACTTCTATTGAGATGCTTAAGATGGT

1 ....
..

3421
GCGAGCTTTTAATATTTTCTTTCCTCGAAGAATTTCAACATAGTTGGAACGCTTTTTTCT

1 ....
..

3481
TCTGGTCATCAATATTGTTTTATAATTAATAAACATGGAGTCATGACCGGAAAAGAAAGA

1 ....
..

3541
AAGAAATGGATTATTAAATCTTCTTAATTGTCTGGTAAACCATCAAAAGAAATTCTCATT

1 ....
..

3601
TTAGAATTACTAATGCAGCATCATATGTTAGCAACTTATTCCTTCATGCAAAATTATATT

1 ....
..

3661
TTTAATCTATTTGCACTATTCTTTTTCTTTGCTCCTCCCTATTTTTGTTTTCAGCTGACA

1 ....
..

3721
GTTCGAAGATGATTATGAAATTTAGAATCATTTAAAGCTATTCTAGACACTGTACTTCTA

1 ....
..

3781
TGTGGTGGATTTTTGCATATTCTCTCTCTCTCTCTTTTTTTTTTTTCGTACATAAAATCCA

1 ....
..

3841
GGAGGAAATAGGTTGCTCTAATCCCTGGGCAAGATGTGCGCTTGAAAAAGCTACATTTGT

1 ....
..

3901
AGCGTTCGAGATTAGCACCAACGAGGCTCAAACCCAAGCGCATTTCATAGTAGTTAATTG

1 ....
..

3961
CAGCCACGAGCCAGCTCAGCCCATTGAGCCACCCCACGTTGGCTGTATTTCCTCTTTGTT

1 ....
..

4021
AAACAACAGCATGGTTATCAAAGGCTTGTGTGTTTGTGTCTTTATGAGTACGCGGGTTTG

1 ....
..

4081
GGGGGAGGGGGATACACAATTTTTAAGTATCATGTTTAGCTTTACAACCATTATTTTCTG

1 ....
..

4141
CGCTTGCTTTTCTTCTTCACGTCCTTAACTTCTCCCCTAGGAGGGCAACTTCAAACTTTA

1 ....
..

4201
TATTTATTTTACAACTTTGCTGTACTTTGTTTCAGGTGTTTTGGACCTTGCTATCAACTT

1 ....
..

4261
CCCAGGATATCATAGATGGAGGTTCACTGACGTTTTGAGAAACGTTCTGAAGATAGTAGT

1 ....
..

4321
TAGTATTGCATGGGTCATCATTCTTCCTCTATTCTATGTGCGTGAACTCTCTTTTGTCCC

1 ....
..

4381
TGAGAACGTTAAAGATATGCTATCGTTTCTTAATCAAGTAAAGGGCGTTTCTCCGCTATA

1 ....
..

4441
TGTCATGGCTGTGGCGCTATACTTGCTCCCAAATTTACTGACAGCTGCTCTGTTCATTTT

1 ....
..

4501
TCCAATGCTCCGGCGTTGGATTGAAAACTCAGATTGGCACATTATTAGGTTACTGTTATG

1 ....
..

4561
GTGGTCACAGGTACTGAATATGTTCCATTTCCATATAGTTCATTGCTACGTTCTTATATC

1 ....
..

4621
ATATAAGAAGCATGAACTTGAAATCAGATATAGTCATAACGATTAATATAGAATGGCTTT

1 ....
..

4681
CACGTATCAGTTAGCGAAAGACCTGAATGTTGAGAAAAGCATATGAATTGAACTACTATC

1 ....
..

4741
TTAAGCTCAGTATATGTCATAGAGTCTAGTATTATCAATCTTAAGAAAGCTACTCTTAAT

1 ....

4801
CTTATATTTCTAGTCTCACAAAATTCATCATGTTGTCTTTACAATATTTGGTTATTCATC

1 ....

4861
TTACTGATATGAATTTAAACCTACTTTTCTTTTTTACTGTTTTTAAATGAGATATGAATA

1 ....

4921
GATAAAAAAATTTATATCATCAAGCTATACCATCAATTGTTTTGTGCCATCTCTTGATCG

1 ....

4981
GGTTTCCCTGTGTGCAGCCTAGAGTTTATGTGGGGAGGGGAATACATGAAAGTCAGTTTG

1 ....

5041
CGCTTATAAAGTAAGTACGAAAATAGATACACTACCTTGCGCCTACCTTGCGCCATGTAA

1 ....

5101
CAACACTTTCCAGTTATGTATTCCTTTTTTCAGTCGGTTGAAATTTCATTAAGTTTAAAT

1 ....

5161
ATATTTCTCTTTCAGGTATACTCTGTTTTGGTTAATACTTTTGTGTGCCAAGTTTGCATT

1 ....

5221
CAGCTATTTTGTCCAGGTGGTCTTGGTTGTTCCTTTTATTTTGCCATTCAAAATAGAGAG

1   ....
..

5281
TTCAGTCGTTGATTTCTATTTTAACCCCCTGCTCATTTTTCCTTCAGATAAAACCACTGG

1   ....
..

5341
TGCAGCCAACAAAAGACATAATGAGCATTCATCGTGTTAAATATGCATGGCATGAATTTT

1   ....
..

5401
TCCCAAATGGTGCTTCTTTTTTTTTCTTCCTTTTATTAGAATAAAATTTCTATTGTCTGC

1   ....
..

5461
TTTATTTTTATTTTTCTGTTAATGGCTATCTTTTCATTTCAGCTGAGAACCACTTGGGAG

1   ....
..

5521
TTGTGTCACTTTGGGCACCGGTAGTGTTGGTCTGTTACTATTTAGACCCTTTTTTATCCT

1   ....
..

5581
TTCTATCAACTTACTATAGCATATCTGGTGAAAATGTCTTGTTACTTTTGGAATTTCTTG

1   ....
..

5641
CAGGTTTATTTTATGGACACTCAAATTTGGTATTCTATTTTCTCAACCATATATGGTGGC

1   ....
..

5701
GTTAGTGGGGCTTTTGATCGCCTTGGAGAGGTAATCCTTTGGAAACCATTTTGCTTTTAT

1 ....

5761
TTTAGAACTCCTTTCCCATCTTCTTTCTTGATCTACATTCACGTACTGTCCATGATTGTT

1 ....

5821
GGACATCATTTATCTGATTAGCTTATTTGCCCTTTACTTAATGTCCATTCTTTTCTGCAA

1 ....

5881
AAACCGAGACACTGACCACGGCTAGATTGTCGGGCATCAAGTAGCGATACTTGAGATAAA

1 ....

5941
TTATCTGTTTGGCAAAATTTGTTTTTAGCTCTCTGTCGAATGGATGGACAATGAACCCAA

1 ....

6001
TTTTGAGAGACTCAACCTGGTTATTAGGCCTTGCCTCCCACATGAATACTTTTTCCTATG

1 ....

6061
TTGTACTAGAATACGTTTTCCTATGACTCGAAACAATCATTAGTTTATAATTTCACTGCA

1 ....

6121
GCCATGCAAGTTGCTTATGGTGTTTTTGGAGTAACTTTTGTAACTGGTCACTTCAAGAAC

1 ....

6181
TGGCTTGTTAATATGCACCAACTTTACAACACTCCCACACAAAAAGTAAAGAGTTATTTG

1 ....
..

6241
TTCTTTCACTCTTCTTTTCTTCAGATAAGAACTTTGGGCATGCTAAGATCACGGTTCCAG

1 ....
..

6301
TCCCTGCCAGGTGCATTTAATGCATACTTGGTACCTACTGATAAATCGCGGAAGAGAGGA

1 ....
..

6361
TTCTCTTTATCAAAGCGATTTGCTGAGGTTAAACTTCTACTGACTCATTATATTGTTGTA

1 ....
..

6421
CAATGACATAAGCGATCATAATAACATCGACTAATGTTTGAATCATTGTCATGCACAGGT

1 ....
..

6481
AACAGCAAACAGAAGAAGCGAAGCTGCAAAATTTGCTCAGCTATGGAATGAAGTAATTTG

1 ....
..

6541
TAGTTTTCGTGAAGAAGACCTAATTAGTGACAGGAAAGTTCCCTTCCATAGATGTTTTAA

1 ....
..

6601
CTCGTATTCAATTTATTATGTTTTACTCTATATGCAGTTAAACTGGGTATTTCTTTAAAA

1 ....
..

6661
CGGGGAGGTGGGCCTTTTACTGGTTCCTTACAAGGGTTTAAATGTATTTTAGTTTATTAT

1 ....
..

6721
GCTTTACTCTACATGCAGTTCAACTAAAACTGTGTATTCTTTAAAACAGGGAGATGGACC

1 ....
..

6781
TTTTACTGGTTCCTTATACATCAGATCCTAGCTTGAAACTGATTCAGTGGCCACCATTTT

1 ....
..

6841
TGCTAGCAAGCAAGGTCCGCAAAGTCCCTTAAATTCGCATTTGCTTTAGTTGCTTTAGTT

1 ....
..

6901
TGTGTTGACTCTATGTCTCCTTCTACAGATCCCAATTGCATTGGACATGGCAGCTCAGTT

1 ....
..

6961
CCGTTCGAAGGACTCTGAACTTTGGAAGCGCATCTGTGCTGATGAATACATGAAATGTGC

1 ....
..

7021
TGTAACTGAATGCTATGAGTCTTTCAAACTTGTTCTAAACACTCTGGTGGTTGGAGAGAA

1 ....
..

7081
TGAGAAAAGGTTGCTAATTCTGTATTGATCTTAGTTTAACTTTTAAGGTTTTCCTCATGA

1 ....
..

7141
TTGATGTTGTTGCATTTTGAAACCATTAAATTCTGTTTAGTGTGTTAAATTATTCAGATT

1 ....
..

7201
CAGTGCTAGTATTTCTGATTGTACGTACCTATAATGTAACAGGACCATAAGAATTATTAT

1 ....
..

7261
CATGGAAATCGAGAGTAACATTTCGAAGAATACTCTTCTTGCAAATTTCAGAATGGCTCC

1 ....
..

7321
TTTACCTGTCCTTTGGAAGAAATTTGTGGAGCTTGTGGGGATCTTGGTCGGTTTGATATG

1 ....
..

7381
AGAAGTAATAAACATAATTATTTCTTCATTTTGCGGTTTTAGTTAATACCTTTTTTATCT

1 ....
..

7441
TTTATTTATTTTATTGCAGAAAGATGGTGATCCCTCCAAAAAGGATGCTGTGGTTTTCTT

1 ....
..

7501
GCTGCAAGATATGTTAGAAGTGGTGACCCGTGATATGATGGTCAATGAAATACGGTTAGT

1 ....
..

7561
GAACTCTGTTTTGCAGTATATGGAAGAAAATGTTCTCTAGAGGTTGCCTTTTTCTAATCA

1 ....
..

7621
AGCTTAATTCCCCTCCTTTTTTCTTGACATATTGTACAGCGAATTAGTAGAGCTAGGACA

1 ....

7681
TAGTAACAAGGAATCGGGAAGGCAACTTTTTGCTGGCACTGACGAAAAACCTGCTCTAGT

1 ....

7741
GTTCCCTCCCGTGCTAACTGCACATTGGGTAGAACAGGTACATCATGCAATAAAATAAAA

1 ....

7801
TAACGTATATTACTTGAAGCACAAATAATGGCTCTAAGAACTTATATTTGATAGGAATCT

1 ....

7861
TTGACATTTTCTAACAACAAATGTCCCACGTTTTAGATACGACGTCTTCATATCCTTCTG

1 ....

7921
ACAATCAAAGAATCTGGCACTGATATACCATCAAATCTTGAGGCTCGTAGAAGGATTGCA

1 ....

7981
TTCTTTGCAAATTCATTGTTTATGGATATGCCACGTGCTCCTCGAGTTCGTAACATGCTC

1 ....

8041
TCGTTCAGGTATTCTATGTTTGCTTAATGTAGTCATCGAATATATGTTCTGGTTACATGT

1 ....

8101
CTCATGATCCTTTTGAAGGTAAATTTACTTTCTTGGCATACTATTTCCATTGCACATATT

1 ....
..

8161
TTTTGTTTTTTTGGAAGACATTGCACATAAAATTGCTAATGCAGTTCTGACATTTTGCCG

1 ....
..

8221
TGGAAACGTGATTGTGTTCTTCTGTGTCTTAGCATTTGCTGAAGTCTACTCCACCCCCCC

1 ....
..

8281
CCCCCCTTTGAATAATTGAATTCATCTTCTTGCCATTGTCAGTGTCCTGACTCCATACTA

1 ....
..

8341
TAGTGAAGAGACTGTCTATTCGAAAACTGAACTTGAGATGGAAAATGAGGATGGTGTATC

1 ....
..

8401
TATCATTTTCTATCTGCAGAAAATATTTCCAGGTTCGTTATATTGTAAACTTCCATAATA

1 ....
..

8461
TCCTTTCTTGAGATTTCCTTTTTCTTTTAGATAACTTCAATGTTATATCTGTTATTGGCT

1 ....
..

8521
TCGTTTAGATGAGTGGAATAACTTTACTGAGCGACTCAACTGTAAGGAGAATGAGATTTG

1 ....
..

8581
GGAAAATGACGAAAAAATCTTGCAGCTTCGCCATTGGGTCTCCTTGAGAGGACAAACTCT

1 ....
..

8641
GTGCAGGACAGGTGCTTGATGCGTTAGTTTCTCTCTTTAATTCATTATTTCCTATCTTGA

1 ....
..

8701
CTAATTTATCTATTTGTGTATCCCTCTCCCGAATGATCATTGGAACTGCAGTCAGAGGAA

1 ....
..

8761
TGATGTATTACAGACGAGCTTTGAAAATTCAGGCTTTTCTCGACATGGCTACTGAAAATG

1 ....
..

8821
GTATGTTCTCTGCAAAGTGTTTAATACAGTTTTTGTGGATTTTAATAGCCAGACTCAGAG

1 ....
..

8881
GTCTTTTCAGGTCGTTCGTCTTCCATGCAAGCTGATTATAATGAGAATAGGGAAATTATG

1 ....
..

8941
CTGATATTATTAGTTTAGTTGATTTGAGTACTTATTCAATAGCACGTGGTAAGCACCAAA

1 ....
..

9001
TACCCTGACCTGTATTGGGTAACTCCTATAACCAACTTTAGTGACCAAAAAAGTAAAACT

1 ....
..

9061
ATGGACAAGGAAATCTATTGTCAATTATTAGATATCTGTCTCGCTAGAATTTGCATGTCT

1 ....
..

9121
GGGATTCCAGTTCCTTACTCCCTCACTAGCCGCTTTTAACGTTTTACCATGTTAGCTACT

1 ....
..

9181
AATATCATTAGACAGACAACGTATTCAGTATTCACTCATAAATAACTCAGAAAATACAAT

1 ....
..

9241
AGCTTGCTCAAGTATCATTATAATAGAACTTTTAAATAAGAATTACAGTAGTGATAAATT

1 ....
..

9301
CCGCCTAATTATTCGATTGGAAACTGCTCTCTTCTGCCATGTATAGATGATAAAAGAAAG

1 ....
..

9361
AAAAGGAAACCAGTAAATTTTATACATTGATTTCTGAGTGTTTGGTGTCTTCACTCTTAG

1 ....
..

9421
TCCCTTAATTAATTCTATGCGTAATTGCTATTTTCCAGTATTTATATTTTCTTTACGAAA

1 ....
..

9481
ACATTGTTGTCAACATGAAATTCCAGAATCGTAATTCCTATCCTTTTAAGTTTTTTGGAT

1 ....
..

9541
AATTCTTAAACCATAAAGCCTAAACCAACCCTTAATTCCTAAACCTAAAATCTTAATCCC

1 ....
..

9601
GAAATCTTAAATTCTATTTTTTAAACCCTAAACTCCAATATAAAACCCTGATCCCAAAAC

1 ....
..

9661
CATAAAACGTTATTCCTTAACCCTAATATCTAATTATTAAACCACTATTCATAAATCCTA

1 ....
..

9721
ATTTTTAAACCATAAACCCTATTAAGAACTCTACTTCCTGATACATAGATCCTAGTTCCT

1 ....
..

9781
TAACCCTTAACCTTTTAACCTTGACTCTTGTCTGAGTAGAAATATTAGAAGGATACAAAG

1 ....
..

9841
CCATCTTAACTGCATCTGATGAAGATAAGAGAAGTCAGAAATCCCTGTATGCCCAGTTGG

1 ....
..

9901
AGGCAGTGGCTGATCTTAAATTTACTTATGTTGCTACCTGTCAAAACTATGGAAATCAAA

1 ....
..

9961
AAAGGAATGGAGACCGTCGTGCAACTGACATCCTGAATTTGATGGTTAAGTAAGTACCCA

1 ....
..

10021
ATCTAATAATATATTTCTTTAGGTCTTTTTGTTTGACATCTTGAACTCTCTCCCTTTCTA

1 ....
..

10081
ACTCCTTTTTCTTATACAGTAATCCCTCGCTTCGTGTGGCATATATTGACGACGTTGAAG

1 ....
..

10141
AAAGGGAGGGTGGAAGAGCACAGAAAGTTTACTATTCTGTACTGGTTAAAGGTGTTGATA

1 ....
..

10201
GTCTTGACCAGGTATCCATCCTTATAAACTGCATATAATAACCTTGTGTCATTACAGTGC

1 ....
..

10261
CACCATAATTGCTAACTAGTGTTTTTATCTTCTTTGAAGGAAATCTATCGGATAAAGTTG

1 ....
..

10321
CCAGGAAATGCAAAGTTAGGAGAAGGAAAACCTGAAAATCAGAATCATGCTTTAATATTT

1 ....
..

10381
ACTCGTGGAGAAGCTCTTCAAACCATTGATATGAATCAGGTAAGGCCATAAAGGCTTGTG

1 ....
..

10441
ATCATCCAAGTAGTAACGGTTTTTTGCAGTAGATCGAATGACATTTAGCGCCAATATTTT

1 ....
..

10501
TCAGGACAATTACTTGGAAGAAGCATTCAAAATGCGTAATCTTTTGGAAGAATTTAATGA

1 ....
..

10561
GGATCATGGAGTAAGACCACCTACAATTTTAGGAGTTCGAGAGCATATCTTTACGGGGAG

1 ....
..

10621
GTCTGACTGAACATTAATATTTGCTTATTTGGTTATTTGCTTGGTCTTGTTTGTCATATC

1 ....
..

10681
TGCTAACTACTTATGTGAACATCTGGGGGTTTAACTTTTATGCTTTGGTATCGCTATTAT

1 ....
..

10741
GCAGCGTCTCTTCTTTAGCTTGGTTCATGTCAAATCAAGAAACAAGCTTTGTCACCATTG

1 ....
..

10801
GTCAAAGAGTTCTTGCAAGACCACTAAAGTATGTTTTCATCTTACTTTCTTCTTCCTATA

1 ....
..

10861
ATCGAATCAAGGCTATTATGTCATGACAAAAGCGCTATCAACAATTATTTATTTATTTTT

1 ....
..

10921
TAAACTATATTGCCGAGAGAATACTTTATGATACGAATGGGAGTGTATTTTCATTTATAA

1 ....
..

```
10981
CAGGTAATAATTATACTCGATATATTGTTAGCATATAAGTGGTAGATACGACAACTTTAA

1     ..........................................................
..

11041
AAATAAATTAAGCACCCTATCCCAACCTTCATCCCTTTGTCTGTAGTTTTACTTTATAGG

1     ..........................................................
..

11101
ACAGCAAGCCACGCCGAAACCACCGATATGAGCTATATTGAGTTTCTTACGGATTGTTTA

1     ..........................................................
..

11161
ATGCAAGTGATCTCGAAGTTGGTTCCAGTGTATGATTTGATATACTTAGAAAAAAAAATT

1     ..........................................................
..

11221
ATTGGTGGGTTTTAGGGTTCGTTTCCATTATGGTCATCCAGATGTGTTCGATAGAATCTT

1     .....................................................ATGA
CAT
                                                  * *****
11281
CCATATAACCCGTGGAGGCATCAGCAAGGGTTCTCGTGGCATCAACTTGAGTGAGGACAT

8
TTCG..TGGCAATATTTTCCAGGACAATTACTTGGAAGAAGCATTCAAAATGCGTAATCT
      *   *** ***  * *  *  ** ***    *    * ****    * **   *
11341
CTTTGCTGGTAATGATCTT.AAATCAGTTAAACT.ACTGTGTCTTCAGTGAGTTTATATT

66
TTTGGAAGAATTTAATGAGGATCATG...GAGTAAGGCCACCTACGATTTTAGGAGTTCG
     * *  * **** ** *    *  *     ***       *   ***** ** *
11399
TATATATGAATATACTATCTTTTCTTTTTATCTAATATTTATTGTACTTTTAACAGGTT.

123
TGAGCACATCTTTACGGGAAGTGTTTCTTCTTTGGCTTGGTTCATGTCAAATCAAGAAAC
     * * * ** ** *  *  ** *    **  *  * **  *   ***  *  ****
```

11458
TTAACTCAACTCTGAGACGAGGGAACATTACTCATCATGAATATATTCAGGTTGGGAAAG

183
A.AGCTTTGTCACCATTGGTCAAAGAGTTCTTGCAAGACCACTCAAGGTTCGCTTCCATT
       **   ***   *   ****   ***   * *   ** * *   *
11518
GTAGGGATGTTGGGTTAAACCAAATTTCACTTTTTGAAGCGAAAGTGGCTTGTGGTAACG

242
ATGGTCATCCAGATGTGTTCGATAGA.ATCTTCCACATAACCCGTGGAGGCATCAGCAAG
       * ** **   ** * *   *   * * *   *   *
11578
GGGAGCAGACACTCAGCAGAGACATCTACAGATTAGGCCATCGATTTGACTTTTTTCGCA

301
GGTTCTCGTGGCATCAACTTGAGTGAAGACATCTTTGCTGGTTTTAACTCAACCCTGAGA
      * * ** ** *   * * ** *   * * *   * * *   ** **
11638
TGCTGTCCTGCTACTTTACT.ACTGTTGGATTTTATTTCAGCTCAATGGTAATTCTTTAT

361
CGAGGGAACATT..ACTCATCATGAATATATTCAGGTTGGGAAAGGTAGGGATGTTGGGT
     *   * ** *** **** * ** *** ** * * *   **   * ***   **
11697
CCTTGCAAGATTCAACTCGTGATTAATCTAATTAAATAAAAGTAGAGTAGATTGTCTAGT

419
TAAACCAA..ATCTCACTTTTTGAAGCGAAAGTGGCTTGTGGTAACGGGGA...GCAGAC
      *   ** *** * *** **** * ***   *   *   *   * ***
11757
CATTTAAATCATCATAACTTTGGAAGTGCCAGTCCATGAAAACATTAAACATATGGAGAG

474
ACTCAGCAGAGACATCTACAGATTAG...........GCCATCGTT........TTGACT
      * * *** ** * ** ******   * ** ***   **   *
11817
TCCCTGCAATGAGAGCTGAAGATTAATTAGAAAGTTAGTAATTGTTAGGTTTTTTTTTT

515
TTTTCCGCATGTTGTCCTGCTACTTTACCACTGTTGGATTTTATTTCAGCTCAAT.....
     ****   *   * * ** *** ** ** * *   * ** * *   *
11877
TTTTTTTGACTCTTTTATGTTACCTTTTCAGAGCTAATGTGAATCCCTTCCTGTTATTGT

570 ...GTTGGTTGTCTTTACAGTCTACTTTTTCCTGTATGGAAGACTTTATTTGTCATT
GAG
       **************************** *************************
**

```
11937
GCAGTTGGTTGTCTTTACAGTCTACTTTTTCTTGTATGGAAGACTTTATTTGTCATTAAG

627
TGGTTTAGAGGAGGCAATACTGAAGTATGCTTCAGCTAGGGGAAATAATTCTCTAAGGGC
     ********** ********** **************** ******* ******* * ***
11997
TGGTTTAGAGCAGGCAATACTTAAGTATGCTTCAGCTAAGGGAAATGATTCTCTTAAGGC

687
GGCCATGGCTTCACAGTCTATAGTTCAATTAGGTATCTTAACTGTACTACCCATGGTCAT
      *********** ********************  * *****
*****************
12057
AGCCATGGCTTCGCAGTCTATAGTTCAATTAGGCGTTTTAACAGTACTACCCATGGTCAT

747
GGAGATTGGATTGGAGAGAGGATTTAGAACTGCATTAGGTGACATCATAATCATGCAGCT

************************************************************
12117
GGAGATTGGATTGGAGAGAGGATTTAGAACTGCATTAGGTGACATCATAATCATGCAGCT

807
TCAGTTGGCATCCGTGTTCTTCACTTTCTCCCTTGGAACAAGAGTCCATTATTTTGGGCG
     ************ **************************************
********
12177
TCAGTTGGCATCTGTGTTCTTCACTTTCTCCCTTGGAACAAGAGTCCATTACTTTGGGCG

867
CACTATTTTGCATGGTGGGGCTAAATACAGAGCAACAGGGCGTGGTTTTGTGGTGCGACA
     ************************************ ********************
**
12237
CACTATTTTGCATGGTGGGGCTAAATACAGAGCAACGGGGCGTGGTTTTGTGGTGCGGCA

927
TGAGAAATTCGCAGAGAACTACCGATTGTACTCAAGGAGCCACTTTGTAAAAGGGCTGGA
     ************************ ***********
***********************
12297
TGAGAAATTCGCAGAGAACTACCGCTTGTACTCAAGAAGCCACTTTGTAAAAGGGCTGGA

987
GCTAATGGTATTGCTTATATGTTATAGGCTATATGGTTCTGCAGCAGATGATGGTATCTC
     ************************ * * * ***************  **  *   ****
12357
GCTAATGGTATTGCTTATATGTTACAAGATTTATGGTTCTGCAGCAAGTGGAGCCGTCTC
```

1047
TTACGCACTCCTCTCATTTTCAATGTGGTTCTTAGTTTTATCCTGGTTGTTTGCTCCTTT
     *** **
****
12417
TTATGCACTCCTCTCATTTTCAATGTGGTTCTTAGTTTTATCCTGGCTGTTTGCTCCTTT

1107
CCTTCTGAATCCATCGGGATTTGAATGGCAAAAGATAGTAGAAGATTGGGAAGACTGGTC
     ****
*****
12477
CCTTCTGAATCCATCGGGATTTGAATGGCAAAAGATAGTAGAAGATTGGGAAGATTGGTC

1167
AAAGTGGATAAGTTGCAGAGGTGGTATTGGAGTTCCCTCCGTTAAGAGCTGGGAATCTTG
     ********* *****************************
****
12537
AAAGTGGATTAGTTGCAGAGGTGGTATTGGAGTTCCCTCAGTTAAGAGCTGGGAATCTTG

1227
GTGGGAGGAAGAACAGGAGCACCTGCGCCATACTGGATTTATAGGACGTTTCTTTGAGAT
     ****************************** ** ** ***** *** ** * **** **
12597
GTGGGAGGAAGAACAGGAGCACCTGCGCCACACCGGGTTTATGGGATGTCTTGTTGATAT

1287
TATACTTTCAATACGCTTTTTTATTTACCAGTATGGAATTGTGTATCATCTAAACATGAC
     *** ***** ******** **
****
12657
TATTCTTTCTATACGCTTCTTCATTTACCAGTATGGAATTGTGTATCATCTAAACATGAC

1347
CACCAGTAGCAGACAAGGTATTCGGCTTAGCATTGTGGT...TTATGGTCTTTCCTGGTT
     ******** ***************** ***********   * * ** *   *
12717
CACCAGTATCAGACAAGGTATTCGGCAGAGCATTGTGGTGAGTATTAGTATCAGTCCATC

1404
GGTCATTGGTGC..TGTGTTGA..TTATTTTGAAGATAGTGTCGATGGGGAGAATGAAGT
      *   * *   * * ** * ** **** * * **   * **   * * * ***
12777
AGCTTCTAATCTAATATCTTAACTTTCTTTTAAGGTTAAAAGCCATTTTCAAATTTTAGT

1460
TCAGTGCGGATTTCCAGTTGATGTTCAGACTTCTTAAGCTATTACTGTTTATTGGGTGTA

```
       ** *  * ***  *****      *     ** * ***     *   *  *
12837
CTTGTACTAAATTCTCTTTGATTAATCTAACATACAATCATTTAGGAGAAAAAAGACCTC

1520
TAGTCACCATTGCAATGTTGTTTTATTTCCTTAATCTCACAATTGGAGATATCTTCCAGA
      ** *   **  **  * **  *  * *     **   **  * **     *
12897
AAG..ATTTTTTACATCCTATTGCTTCCCTTAGGATGCATGCGTGACGGTAATGCTTATT

1580
GCATACTGGCCTTTATGCCGACAGGGTGGGCTCTTCTGCAGATATCACAAGCATGTCGAA
        *    *     * ** **        *    *  * **    *** * ***
12955
AAGGATGAATCCCATGGTAGATAGCAAACAGAGTAGACTAATTGTCGTGT.CATTTGGAA

1640
CACTGGTGAAGGGAATAGGA.ATGTGGGGGTCAGTAAAGGCACTAGCAAGAGGGTATGAA
      * * ****   * *  * * ** *   ** ****     ** **  * * * **
13014
AA..GATGAAAATATTTCTACAAGTTGAATTCTGTAACATTGTTA.CATAATTGAAAGAT

1699
TACATGATGGGTGTGTTACTGTTTGCAC.CAATAGCTATATTGGCATGGTTCCCCTTCGT
        ** *    * *    * ** *   *  *  ** **   * *** *  **
13071
CTTGTGTTTAAGGGTTGCTAATCTGTAAATACCAAGAATGTTCATAAAGTTGCTTTTTTG

1758
CTCAGAATTCCAGACCAGGCTGCTATTCAACCAAGCTTTCA..GCCGAGGCCTCCAAATC
      **   ** **        ** * *   *  *   *   **** *     *  *
13131
TTCTTGGTTTTAGGTTTATTGTCTTTCCTGGCTGGTCATTGTTGCCGTGTTGATTATTTT

1816
CAACGTATTCTGGCTGGCAGCAAGAAGCAAGCCTAA........................
      ** **         * **  **   *  * **
13191
AAAGGTGAGACTCGAAACCCCATAAAATCATTCCAAAACGAGAATCATCCCTTGGTTGGC

1852 .........................................................
...

13251
TTTCTAAAAGAAACTGACGCCACTTTTTAAATTTGCAGATAGTGTCCATGGGTAGGATGA

1852 .........................................................
...
```

13311
AGTTCAGTGCGGATTTCCAGCTAATGTTCAGACTTGTTAAGCTAGTCATGTTTGTGGGGA

1852 ....
...

13371
GTATAGTCACTATTGCAATGCTGTTTTATTTCCTTGATCTCACAATTGGAGATATCTTTC

1852 ....
...

13431
AGAGCATACTGGCCTTTGTGCCGACAGGGTGGGCTCTTCTGCAGGTACATGTTGGCGCTA

1852 ....
...

13491
ATTATTTGTCATTTCTATACTCTATCAATGGATGAACATTGGGTTTAATTTAGTTGTGTA

1852 ....
...

13551
AATGTTGATGAATGATCAGATATCACAAGCATGCCGGACAGTGGTGAAGGGAATAGGAAT

1852 ....
...

13611
GTGGGGGTCAGTGAAGGCACTAGCAAGAGGGTACGAATACATGATGGGGGTGTTGCTGTT

1852 ....
...

13671
TGCACCAATAACTATATTAGCATGGTTCCCCTTCGTCTCAGAATTCCAGACCAGGCTGCT

1852 ....
...

13731
ATTCAACCAAGCTTTCAGCCGAGGCCTTCAAATCCAACGAATTCTGGCTGGTAGCAAGAT

1852 ....
...

13791
GCAAGCCTAAAAAAATACGATTGTTGTTTTATGAACTTCGGCTTTTACACGAATTTGATT

1852 ....

13851
TTGGAAAGAAGTAAGATTAGCTTTCAACAATATGATTCCTCTAACAAGATTACTTAACAC

1852 ....

13911
CTCACTTGTAATATTAAATGCTTAAGTTTGGTTCAGAAAGTCAGAATGTTCGATTCTTGT

1852 ....

13971 GTTTAGTGCATGAATGTAAGATTGTTTACCAAAT
