# Supplementary material for: GhCalS5 is involved in cotton response to aphid attack through mediating callose formation
Source: Front Plant Sci. 2022 Jul 20;13:892630. doi: 10.3389/fpls.2022.892630 (PMC9350506; doi:10.3389/fpls.2022.892630)
Supplement: Supplementary file 7 [file Data_Sheet_7.PDF]

Alignment of GhCalS5(upper line) and GhCalS5.1(lower line)
Identity=43.43%(800/1842) Gap=45.37%(1530/3372)
----

1 ....
..

1
CGCTTTTGCATGGGACAACTCCCTTCCCTCTCTTTTTTTTTCTGCTTTTCGGATTAAACG

1 ....
..

61
ATTTTGAGCAGTAGAGAGTTGAGAAGATAACGAGTTAACTCAGTGCGGGATGACGAATAC

1 ....
..

121
CGAACCGGGTGCGGGAGCATCATCAACGCAAGGGCTGACAAGGCGGCCGTCACGGAGCGC

1 ....
..

181
TGCAACCACAACGTTCTCAACGGAGGTGTTCGACAATGAGGTCGTACCTTCCTCACTTAG

1 ....
..

241
TTCCATCGCTCCTATCCTCCGCATTGCCAAAGAGATCGAAACTGAACGCCCTCGTGTCGC

1 ....
..

301
CTATCTCTGCCGTTTCTATGCTTTTGAGAAAGCACACAGGTTAGATCCCCACTCCTCCGG

1 ....
..

361
TCGTGGAGTAAGGCAATTCAAGACTGGTTTGCTGCAAAGATTAGAGAGGGACAATGCATC

1 ....
..

421
CAGTCTTGCCTCTCGGGTTAAAAAGACAGATGCTAAGGAAATTGGGAGCTACTATCAGCA

1 ....
..

481
GTACTATGAACACTATGTTAGAGCATTGGACCAAGGAGACCAGGCAGACAGAGCTCAACT

1 ....
..

541
AGGGAAAGCTTACCAAACAGCCGGGGTGCTTTTTGAAGTGCTCTGTGCCGTTAATAAGAC

1 ....
..

601
TGAGAAAGTTGAAGAAGTTGCTCCCGAGATTATGGCAGCGGCCAAAGATGTCCAAGAAAA

1 ....
..

661
GAAGGAAATCTACACTCCTTACAACATTCTTCCTTTGGATGCTGCCGGAGCTTCTCAGTC

1 ....
..

721
TATTATGCAGCTTGAGGAGGTTAAGGCTTCTGTGGCTGCACTAGGGAATGTTCGTGGCTT

1 ....
..

781
GAACTGGCCTTCGGGATTCGAGCCACAAAGGCAAAAAACCGGAGACTTGGACCTTCTGGA

1 ....
..

841
TTGGTTAAGAGCCATGTTTGGATTCCAGAGGGACAATGTCAGGAACATGAGGGAGCACTT

1 ....
..

```
901
GATTTTGCTGCTTGCCAATAATCATATAAGACTCCACCCCAAGCCTGAACCTCTTAATAA

1     ..........................................................
..

961
GCTTGATGAACGAGCAGTCGATGCTGTTATGAGCAAGCTTTTTAAGAATTACAAAACATG

1     ..........................................................
..

1021
GTGTAAATTTTTAGGACGTAAACATAGTTTAAGGCTGCCTCAAGGCTCTCAAGAAATACA

1     ..........................................................
..

1081
ACAAAGGAAGATACTATATATGGGATTATATCTTCTCATCTGGGGTGAAGCAGCTAATGT

1     .........................................................A
TGA
                                                       ***
1141
TCGATACATGCCGGAATGTCTATGTTACATTTTTCATAATATGGCATATGAACTCCATGG

5
CATTTCG..TGGCAATATTTTCCAGG..ACAATTACTTGGAAGAAGCATTCAAAATGCGT
     * * * *  *** *** **  *   *  **         *  **** **** *  * *
1201
CCTGTTGGCTGGAAATGTTAGCATAGTGACGGGAGAAAATATCAAGCCTTCATACGGTGG

61
AATCTTTTGGAAGAATTTAATGAGGATCATGGAGTAAGGCCACC.TACGATTTTAGGAGT
     *    *  *     **   ** * * **          **  *    **  *   *
1261
AGATGATGAGGCATTCTTGCGGAAGGTTATAACACCCATTTACTGTGTCGTTGCAAAGGA

120
TCGTGAGCACATCTTTACGGGAAGTGTTTCTTCTTTGGCTTGGTTCATGTCAAATCAAG.
        **  * * *   *  ****  * ****  *   *  ****  *  *   ** *
1321
AGCCGAAAAGAACAAAAATGGAACAGCTTCTCATGCAGACTGGTGTAATTATGATGATCT

179
AAACAAGCTTTGTCA..CCATTGGTCAAAGAGTTCTTGCAAGACCACTCAAGGTT.....
      *** **  **      *   ** *       ***** * * **  *    * *
```

```
1381
GAACGAGTATTTCTGGTCTGCTGATTGCTTCTCTCTTGGATGGCCTATGCGTGATGATGG

232
CGCTTCCATTATGGTCATCCA.GATGTGTTCGATAGAATCTTCCACATAACCCGTGGAGG
      *  *  * *    * *** **   *   * *  *      * **   ***** *
1441
TGACTTTTTCAAATCAACCCATGACACGGGAAAGAAGAGTGGTGCAAGAAAATGTGGAAG

291
CATCAGCAAG...GGTTCTCGTGGCATCAACTTGAGTGAAGACATCTTTGCTGGTTTTAA
     **  ****     ** *   *  ** *      *   ** *****   ***** *
1501
CACGGGCAAATCAAATTTTGTAGAGATTAGAACATTTTGGCACCTCTTTCGAAGTTTTGA

348
CTCAACCCTGAGACGAGGGAACATTACTCATCATGAATATATTCAGGTTGGGAAAGGTAG
       *     **      *   *  *   ** * **  **    ***     ** *
1561
TCGACTATGGACTTTTTATATTCTAGGTTTGCAGGTATTGATCATTATTGCATGGAGTGG

408
GGATGTTGGGTTAAA.....CCAAATCTCACTTTTTGAAGCGAAAGTGGCTTGTGGTAAC
      * *      * *      ***    * ** *** *   *  *   * * * *
1621
AGCTCCGATAACAGAAATCTTTAAAGAGGAATTATTGTATGATATATCTAGTATTTTCAT

463
GGGGAGCAGACACTCAGCAGAGACATCTACAGATTA.GGCCATCGTTTTGACTTTTTCCG
        ***** ** ** **    *  **    ***  ** * *  * * *  **  * *
1681
CAC.AGCAGCCATTCTGCGTTTAGTTCAGAGTATTTTGGACCTTTCTCTCAACTTCCCTG

522
CATGTTGTCC.TGCTACTTTACCACTGT..TGGATTTTATTTCAGC........TCAATG
      ** *  *  ** * ** **    ** **       **          *** *
1740
GATATCATAGGTGGAAGTTCACTGACGTATTGAGAAACGTTCTGAAGATAATAGTCAGTA

571
TTGGTTGTCTTTACAGTCTACTTTTTCCT...GTATGGAAGACTTTATTTGTCATTGAGT
     *** ***  *  *  *** * * *     **  *  *  **  ******  * **
1800
TTGCTTGGGTCATCGTTCTTCCTCTCTTCTACGTGCGTGAATTTTCTTTTGTCCCTCAGA

628
GGTTTAGAGGAGGCAATACTGAAGTATGCTTCAGCTAGGGGAAATAATTCTCTA...AGG
       *** ****    **       *  * **  * *** * **** *****   *
```

```
1860
ACGTTA.AGGATATGCTATCATTTCTTAATCAAGTAAAGGGGATTAATCCTCTATATATC

685
GCGGCCATGGCTTCACA...GTCTATAGTTCAATTAG..GTATCTTAACTGTACTACCCA
       ***  ***    * *   *  *  *  ** * * *  *  * *    *   **  ***
1919
ATGGCTGTGGGACTATACTTGCTTCCAAATCTACTGGCAGCTTTTCTGTTCATCTTTCCA

740
TGGTCATGGAGATTGGATTGGAGAGAGGATTTAGAACTGCATTAGGTGAC....ATCATA
       **   ** * ******** * *      ** * **   ******* **    **  *
1979
ATGTTCCGGCG.TTGGATTGAAAACTCAGATTGGCACATTATTAGGTTACTGTTATGGTG

796
ATCATGC.....AGCTTCAGTTGGCATCCGTGTTCTTCACTTTCTCCCTTGGAACA.AGA
      ***        ** **  ***** *   *  *  * * *   **    *  *   * * *
2038
GTCACAGCCGCGAGTTTATGTTGGGAGGGGAATGCATGAAAGTCAGTTTGCGCTTATAAA

850
GTCCATTATTTTGGGCGCACTA.TTTTGCATGGTGGGGCTAAATACAGAGCAACAGGGCG
     **  * * * **  * * * ** *****  ***   *  *  ** ***      * **
2098
GTATACTCTCTTTTGGGTATTACTTTTGTGTGGCAAGTTTGCATTCAGCTACTTTGTGCA

909
TG...GTTTTGTGGTGCGACATGAGAAATTCGCAGAGAACTACCGATTGTACTCAAGGAG
              ******  *     ***  *  *  ** * *    * **  *  *   *
2158
GATAAAACCATTGGTGCAGCCGACAAAAGACATAATGAGC.ATTCGTCGTGTTAGATATG

966
CCACTTTGTAAAAGGGCTGGAGCTAATGGTATTGCTTATATGTTATAGGCTATATGGTTC
     *       ***    *   *        *   ***    * *** *  *   *
2217
CATGGCATGAAATTTTCCCTAATGCTCAAAACAACTTGGGAGCTATTGTGTCACTTTGGG

1026
TGCAGCAGATGATGGTATCTCTTACGCACTCCTCTCATTTTCAATGTGGTTCTTAGTTTT
       * *    *  **** * * *** * ** * **  ****   **    ** *
2277
CACCGGTTGTATTGGTTTATTTTATGGACAC.TCAGATTTGGTATTCTATTTTCTCAACC

1086
ATCCTGGTTGTTTGCTCCTTTCCTTCTGAATCCATCGGGATTTGAATGGCAAAAGATAGT
     **    * ** ** *     * *** ***   * * **      **   *    *  *
```

```
2336
ATATCCGGTGGTTTCAGTGGTGCTTTTGATCGCCTTGGAGAGATAAGAACTTTGGGCATG

1146
AGAAGATTGGGA....AGACTGGTCAAAGTGGATAAGTTGCAGAGGTGGTATTGGAGTTC
       *****  *    ** *    *  *** **   ****    ****
2396
CTAAGATCACGGTTCCAGTCCTTGCCTGGTGCATTTAATGCATGCTTGGTGCCTACTGAA

1202
CCTCCGTTAAG.AGCTGGGAATCTTGGTGGGAG.GAAGAACAGGAGCACCTGCGCCATAC
        **    * ** *    **** *   ** **   * *  * * ****    *
2456
AAATCGCGGCGTAGAGGATTCTCTTTATCAAAGCGATTTGCTGAGGTAACTGCAAACAAA

1260
TGGATTTATA..GGACGTTTCTTTGAGATTATACTTTCAATACGCTTTTTATTTACCAG
      * * * *   * *   *** * ** ****    * ** *  ** *   ***
2516
AGAAGTGAAGCTGCAAAATTCGCTCAG.TTATGGAATGAAATCATTTGTAGCTTTCGTGA

1318
TATGGAATTGT...GTATCATCTAAACATGACCACCAGTAGCAGACAAGGTATTCGGCTT
        **  *    *** **   * *    *     ***     *  **  *
2575
AGAAGACCTAATTAGTAACAGGGAGATGGACCTTTTGCTAGTTCCTTATACATCGGATCC

1375
AGCATTG....TGGTTTATGGTCTTTCCTGGTTGGTCATTGGTGCTGTGTTGATTATTTT
        ***    ***** * * *   * * *** *     *    *   **    **
2635
TAGCTTGAAAATGGTTCAGTGGCCACCGTTTTTGCTGGCAAGCAAGATCCCAATAGCATT

1431
GAAGATAGTG..TCGAT........GGGGAGAATGAAGTTCAGTGCGGATTTCCAGTTGA
     * * ** *   ** **         ***   ** **  *    * *    * ***
2695
GGATATGGCAGTTCAATTCCGCTCCAAGGACGCGGACCTTTGGAAGCGCATCTGTGCTGA

1481
TGTTCAGACTTCTTAAGCTATTACTGTTTATTGGGTGTAT.....AGTCACCATTGCAAT
     **   * *    * *** * * **  *  * *  * *    * *   * *  **
2755
TGAATACATGAAATGTGCTGTGATTGAATGCTATGAATCTTTCAAAATTGTCCTAAAAAC

1536
GTTGTTTTATTT......CCTTAATCTCACAATTGGAGATATCTTCCAGAGCATACTGGC
      *** *  *       *   **   ** *** ** **** ** *    **  *
```

```
2815
TTTGGTGGTTGGAGAGAACGAGAAAAGGACCATTAGAATTATCATCAAAGAAATCGAGAA

1590
CTTTATGCCGACAGGGTGGGCTCTTCTGCAGATATCACAAGCATGTCGAACACTGGTGAA
        **  *** *** *  ** *  **** ** *** **     *    *** *
2875
TAACATCTCGA.AGGATACTCT.TCTTGCAAATTTCAGAATGGCTCCTTTACCTGTTCTT

1650
GGGAATAGGAATGTGGGGGTCAGTAAAGGCACTAGCAAGAGGGTATGAATACATGATGGG
      * ** *   ***** * *  **  *    *  *   ** **   * **  * **
2933
TGCAAGAAATTTGTGGAGCTT.GTTGGGATCTTGAAAGATGGTGATCCCTCCAAAAAGGA

1710
TGTGTTACTGTTTGCACCAATAGCTATATTGGCA.TGGTTCCCCTTCGTCTCAGAATT..
     **  *  * **   *    ** *** ** * * * *** *** *  * * *  *
2992
TGCTGTGGTTTTCTTGCTGCAAGATATGTTAGAAGTAGTTACCCGTGATATGATGGTGAA

1767
CCAGACCAGGCTGCTATTCAACCAAGCTTTCAGC..CGAGGCCTCCA.AATCCAACGTAT
      ***   *    ** *  * * **   ***  * ***  **  **  **** * *
3052
TGAGATACGCGAATTAGTAGAGCTAGGACACAGTAACAAGGAATCGGGAAGGCAACTTTT

1824
TCTGGCTGGCAGCAAGAAGCAAGCCTAA................................
     *  * *      * ** *  **  *
3112
TGCTGGTACTGATGAAAAACCTGCTATAGCGTTCCCTCCTGAGCTAACTGCTCATTGGAT

1852  .........................................................
...

3172
AGAACAGATACGACGCCTTCATATCCTTCTCACAGTCAAAGAATCTGGCACTGATATACC

1852  .........................................................
...

3232
ATCAAATCTTGAGGCGCGTCGAAGGATTTCATTCTTTGCAAACTCATTGTTTATGGATGA

1852  .........................................................
...
```

3292
AACCGAACCACGTATGTAGCAGATGCCACTAGGGGTGAAGGAGGTTAATAAATTTCAATG

1852 ....

3352 CTTTTTTTAAAA
