# Supplementary material for: GhCalS5 is involved in cotton response to aphid attack through mediating callose formation
Source: Front Plant Sci. 2022 Jul 20;13:892630. doi: 10.3389/fpls.2022.892630 (PMC9350506; doi:10.3389/fpls.2022.892630)
Supplement: Supplementary file 8 [file Data_Sheet_8.PDF]

Alignment of GhCalS5(upper line) and GhCalS5-like(lower line)
Identity=97.73%(1809/1851)  Gap=67.91%(3918/5769)
-----------------

1 ..........................................................
..

1
ATGACGAATACCGAACCGGGTGCGGGAGCATCATCAACGCAAGGGCTGACAAGGCGGCCG

1 ..........................................................
..

61
TCGCGGAGCGCTGCAACCACAACGTTCTCAACGGAGGTGTTCGACAATGAGGTCGTACCT

1 ..........................................................
..

121
TCCTCACTTAGTTCCATCGCTCCTATCCTCCGCATTGCCAAAGAGATCGAAACTGAACGC

1 ..........................................................
..

181
CCGCGTGTCGCCTATCTCTGCCGTTTCTATGCTTTTGAGAAAGCACACAGGTTAGATCCC

1 ..........................................................
..

241
AACTCCTCCGGTCGTGGAGTAAGGCAATTCAAGACTGGTTTGCTGCAAAGATTAGAGAGG

1 ..........................................................
..

301
GACAATGCATCCAGTCTTGCCTCTCGGGTTAAAAAGACAGATGCTAAGGAAATTGGGAGC

1 ..........................................................
..

361
TACTATCAGCAATACTATGAACACTATGTTAGAGCATTGGACCAAGGAGACCAGGCAGAC

1 ..........................................................
..

421
AGAGCTCAACTTGGGAAAGCTTACCAAACAGCCGGTGTGCTTTTTGAAGTGCTCTGTGCC

1 ............................................................
..

481
GTTAATAAGACTGAGAAAGTTGAAGAAGTTGCTCCCGAGATTATGGCAGCGGCCAAAGAT

1 ............................................................
..

541
GTCCAAGAAAAGAAGGAAATCTACACTCCTTACAACATTCTTCCTTTGGATGCTGCCGGA

1 ............................................................
..

601
GCTTCTCAGTCTATTATGCAGCTTGAGGAGGTTAAGGCTTCTGTGGCTGCACTAGGGAAT

1 ............................................................
..

661
GTTCGTGGATTGAACTGGCCTTCGGGATTCGAGCCACAAAGGCAAAAAACCGGAGACTTG

1 ............................................................
..

721
GACCTTTTGGATTGGTTAAGAGCCATGTTTGGGTTCCAGAGGGACAATGTCAGGAACATG

1 ............................................................
..

781
AGGGAGCACTTGATTTTGCTGCTTGCCAATAATCATATAAGACTCCACCCCAAGCCTGAA

1 ............................................................
..

841
CCTCTTAATAAGCTTGATGAACGAGCAGTCGATGCTGTTATGAGCAAGCTTTTTAAGAAT

1 ............................................................
..

901
TACAAAACATGGTGTAAATTTTTAGGACGTAAACATAGTTTAAGGCTGCCTCAAGGCTCT

1 ............................................................
..

961
CAAGAAATACAACAAAGGAAGATACTATATATGGGATTATATCTTCTTATCTGGGGTGAA

1 ............................................................
..

1021
GCAGCTAATGTTCGATACATGCCAGAATGTCTATGTTACATTTTTCATAATATGGCATAT

1 ............................................................
..

1081
GAACTCCATGGCCTGTTGGCTGGAAATGTTAGCATAGTGACGGGGGAAAATATCAAGCCT

1 ............................................................
..

1141
TCTTACGGTGGAGATGATGAGGCATTCTTGCGGAAGGTTATAACACCCATTTACTGTGTC

1 ............................................................
..

1201
GTTGCAAAGGAAGCCGAAAAGAACAAAAATGGAACGGCTTCTCATGCAGACTGGTGTAAT

1 ............................................................
..

1261
TATGATGATCTGAACGAGTATTTCTGGTCTGCTGATTGCTTCTCTCTTGGATGGCCTATG

1 ............................................................
..

1321
CGTGATGATGGTGACTTTTTCAAATCAACCCATGACACGGGAAAGAAGAGTGGTGCAAGA

1 ............................................................
..

1381
AAATGTGGAAGCACGGGCAAATCAAATTTTGTAGAGATTAGAACTTTTTGGCACCTCTTT

1 ..........................................................
..

1441
CGAAGTTTTGATCGACTATGGACTTTTTATATTCTAGGTTTGCAGGTATTGATCATTATT

1 ..........................................................
..

1501
GCATGGAGTGGAGCTCCGATAACAGAAATCTTTAAAAAGGATTTATTGTATGATATATCC

1 ..........................................................
..

1561
AGTATTTTCATCACAGCAGCCATTCTGCGTTTAGTTCAGAGTATTTTGGACCTTTCTCTC

1 ..........................................................
..

1621
AACTTCCCTGGATATCATAGGTGGAAGTTCACTGACGTATTGAGAAACGTTCTGAAGATA

1 ..........................................................
..

1681
ATAGTCAGTATTGCTTGGGTCATCGTTCTTCCTCTCTTCTACGTGCGTGAATTCTCCTTT

1 ..........................................................
..

1741
GTCCCTCAGAACGTTAAGGATATGCTATCGTTTCTTAATCAAGTAAAGGGGATTAATCCT

1 ..........................................................
..

1801
CTATATATCATGGCTGTGGGGCTATACTTGCTTCCAAATCTACTGGCAGCTTTTCTGTTC

1 ..........................................................
..

1861
ATTTTTCCAATGTTCCGGCGTTGGATTGAAAACTCAGATTGGCACATTATTAGGTTACTG

1 ..........................................................
..

1921
TTATGGTGGTCGCAGCCGCGAGTTTATGTTGGGAGGGGAATGCATGAAAGTCAGTTTGCG

1 ..........................................................
..

1981
CTTATAAAGTATACTCTCTTTTGGGTATTACTTTTGTGTGGCAAGTTTGCATTCAGCTAC

1 ..........................................................
..

2041
TTTGTCCAGATAAAACCATTGGTGCAGCCGACAAAAGACATAATGAGCATTCGTCGTGTT

1 ..........................................................
..

2101
AGATATGCATGGCATGAAATTTTCCCTAATGCTCAAAACAACTTAGGAGCTATTGTGTCA

1 ..........................................................
..

2161
CTTTGGGCACCGGTTGTATTGGTTTATTTTATGGACACTCAGATTTGGTATTCTATTTTC

1 ..........................................................
..

2221
TCAACCATATCCGGTGGTTTCAGTGGTGCTTTTGATCGCCTTGGAGAGATAAGAACTTTG

1 ..........................................................
..

2281
GGCATGCTAAGATCACGGTTCCAGTCCTTGCCTGGTGCATTTAATGCATGCTTGGTGCCT

1 ..........................................................
..

2341
ACTGAAAAATCGCGGCGTAGAGGATTCTCTTTATCAAAGCGATTTGCTGAGGTAACTGCA

1 ..........................................................
..

2401
AATAAAAGAAGTGAAGCTGCAAAATTCGCTCAGTTATGGAATGAAGTCATTTGTAGCTTT

1 ..........................................................
..

2461
CGTGAAGAAGACCTAATTAGTAACAGGGAGATGGACCTTTTACTAGTTCCTTATACATCG

1 ..........................................................
..

2521
GATCCTAGCTTGAAAATGGTTCAGTGGCCACCGTTTTTGCTGGCAAGCAAGATCCCAATA

1 ..........................................................
..

2581
GCATTGGATATGGCAGTTCAATTCCGCTCCAAGGACGCGGACCTTTGGAAGCGCATCTGT

1 ..........................................................
..

2641
GCTGATGAATACATGAAATGTGCTGTGATTGAATGCTATGAGTCTTTCAAATTTGTCCTA

1 ..........................................................
..

2701
AAAACTTTGGTGGTTGGAGAGAACGAGAAAAGGACCATTAGAATTATCATCAAAGAAATC

1 ..........................................................
..

2761
GAGAATAACATCTCGAAGGATACTCTTCTTGCAAATTTCAGAATGGCTCCTTTACCTGTT

1 ..........................................................
..

2821
CTTTGCAAGAAATTTGTGGAGCTTGTTGGGATCTTGAAAGATGGTGATCCCTCCAAAAAG

1 ..........................................................
..

2881
GATGCTGTGGTTTTCTTGCTGCAAGATATGTTAGAAGTAGTTACCCGTGATATGATGGTG

1 ..........................................................
..

2941
AATGAGATACGCGAATTAGTAGAGCTAGGACACAGTAACAAGGAATCGGGAAGGCAACTT

1 ..........................................................
..

3001
TTTGCTGGTACTGATGAAAAACCTGCTATAGCGTTCCCTCCTGAGCTAACTGCTCATTGG

1 ..........................................................
..

3061
GTAGAACAGATACGACGCCTTCATATCCTTCTCACAGTCAAAGAATCTGGCACTGATATA

1 ..........................................................
..

3121
CCATCAAATCTTGAGGCGCGTCGAAGGATTTCATTCTTTGCAAACTCATTGTTTATGGAT

1 ..........................................................
..

3181
ATGCCACGTGCTCCTCGAGTTCGTAACATGCTTTCATTCAGTGTCCTGACTCCGTACTAT

1 ..........................................................
..

3241
AGTGAAGAGACTGTTTATTCCAAAAGTGAACTTGAGATGGAAAACGAGGATGGTGTATCT

1 ..........................................................
..

3301
ATCATTTTCTATCTGCAGAAAATATTTCCAGATGAGTGGAACAACTTTATTGAGAGACTC

1 ............................................................
..

3361
AATTGTAAGGAGAATGAAATTTGGGAAAATGATGAAAAAATCTTGCAGCTTCGTCATTGG

1 ............................................................
..

3421
GTCTCCTTTAGAGGACAAACTCTGTGCAGGACAGTTAGAGGGATGATGTATTACAGACGA

1 ............................................................
..

3481
GCTTTGAAAGTTCAGGCTTTCCTTGACATGGCTGATGAAAAAGAAATATTAGAAGGATAC

1 ............................................................
..

3541
AAAGCGATCTTAACACCATCAGATGAAGATAAGAAAAGCCAGAGATCCCTATATGCTCAG

1 ............................................................
..

3601
TTGGAGGCAGTGGCTGACTTAAAATTTACTTATGTTGCTACCTGTCAAAACTATGGAAAT

1 ............................................................
..

3661
CAAAAAAGGAATGGAGACCGTCGTGCAACTGACATCCTTAATTTGATGGTTAATAATCCC

1 ............................................................
..

3721
TCTCTTCGTGTGGCATACATTGACGAAATTGAAGAAAGGGATGGTGGAAAAGCTCAGAAA

1 ............................................................
..

3781
GTTTACTATTCTGTACTGGTTAAAGGTGTTGATAATCTTGACCAGGAAATCTATCGAATA

1     ..........................................................
..

3841
AAGTTGCCTGGAAATGCGAAGTTAGGAGAAGGAAAACCTGAAAATCAGAATCATGCTTTA

1     ...............ATGACATTTCGTGGCA...ATATTTTCCAGGACAATTACTTG
GAA
                    *  ***    **   ****    *****************
3901
GTTTTCACTCGGGGAGAAGCTCTTCAAACCATTGATATGAATCAGGACAATTACTTGGAA

43
GAAGCATTCAAAATGCGTAATCTTTTGGAAGAATTTAATGAGGATCATGGAGTAAGGCCA

************************************************************
3961
GAAGCATTCAAAATGCGTAATCTTTTGGAAGAATTTAATGAGGATCATGGAGTAAGGCCA

103
CCTACGATTTTAGGAGTTCGTGAGCACATCTTTACGGGAAGTGTTTCTTCTTTGGCTTGG

************************************************************
4021
CCTACGATTTTAGGAGTTCGTGAGCACATCTTTACGGGAAGTGTTTCTTCTTTGGCTTGG

163
TTCATGTCAAATCAAGAAACAAGCTTTGTCACCATTGGTCAAAGAGTTCTTGCAAGACCA

************************************************************
4081
TTCATGTCAAATCAAGAAACAAGCTTTGTCACCATTGGTCAAAGAGTTCTTGCAAGACCA

223
CTCAAGGTTCGCTTCCATTATGGTCATCCAGATGTGTTCGATAGAATCTTCCACATAACC
     ***********
************************************************
4141
CTCAAGGTTCGTTTCCATTATGGTCATCCAGATGTGTTCGATAGAATCTTCCACATAACC

283
CGTGGAGGCATCAGCAAGGGTTCTCGTGGCATCAACTTGAGTGAAGACATCTTTGCTGGT
     ***************************************** **
***************
4201
CGTGGAGGCATCAGCAAGGGTTCTCGTGGCATCAACTTGAGCGAGGACATCTTTGCTGGT

343
TTTAACTCAACCCTGAGACGAGGGAACATTACTCATCATGAATATATTCAGGTTGGGAAA

************************************************************
4261
TTTAACTCAACCCTGAGACGAGGGAACATTACTCATCATGAATATATTCAGGTTGGGAAA

403
GGTAGGGATGTTGGGTTAAACCAAATCTCACTTTTTGAAGCGAAAGTGGCTTGTGGTAAC

************************************************************
4321
GGTAGGGATGTTGGGTTAAACCAAATCTCACTTTTTGAAGCGAAAGTGGCTTGTGGTAAC

463
GGGGAGCAGACACTCAGCAGAGACATCTACAGATTAGGCCATCGTTTTGACTTTTTCCGC

************************************************************
4381
GGGGAGCAGACACTCAGCAGAGACATCTACAGATTAGGCCATCGTTTTGACTTTTTCCGC

523
ATGTTGTCCTGCTACTTTACCACTGTTGGATTTTATTTCAGCTCAATGTTGGTTGTCTTT
     ***********************
************************************
4441
ATGTTGTCCTGCTACTTTACCACCGTTGGATTTTATTTCAGCTCAATGTTGGTTGTCTTT

583
ACAGTCTACTTTTTCCTGTATGGAAGACTTTATTTGTCATTGAGTGGTTTAGAGGAGGCA

************************************************************
4501
ACAGTCTACTTTTTCCTGTATGGAAGACTTTATTTGTCATTGAGTGGTTTAGAGGAGGCA

643
ATACTGAAGTATGCTTCAGCTAGGGGAAATAATTCTCTAAGGGCGGCCATGGCTTCACAG
     ********************************************
***************
4561
ATACTGAAGTATGCTTCAGCTAGGGGAAATAATTCTCTAAGGGCAGCCATGGCTTCACAG

703
TCTATAGTTCAATTAGGTATCTTAACTGTACTACCCATGGTCATGGAGATTGGATTGGAG
     *********** **************
*********************************
4621
TCTATAGTTCAGTTAGGTATCTTAACAGTACTACCCATGGTCATGGAGATTGGATTGGAG

763
AGAGGATTTAGAACTGCATTAGGTGACATCATAATCATGCAGCTTCAGTTGGCATCCGTG

************************************************************
4681
AGAGGATTTAGAACTGCATTAGGTGACATCATAATCATGCAGCTTCAGTTGGCATCCGTG

823
TTCTTCACTTTCTCCCTTGGAACAAGAGTCCATTATTTTGGGCGCACTATTTTGCATGGT
***********************************
************************
4741
TTCTTCACTTTCTCCCTTGGAACAAGAGTCCATTACTTTGGGCGCACTATTTTGCATGGT

883
GGGGCTAAATACAGAGCAACAGGGCGTGGTTTTGTGGTGCGACATGAGAAATTCGCAGAG
*****************************************
******************
4801
GGGGCTAAATACAGAGCAACAGGGCGTGGTTTTGTGGTGCGGCATGAGAAATTCGCAGAG

943
AACTACCGATTGTACTCAAGGAGCCACTTTGTAAAAGGGCTGGAGCTAATGGTATTGCTT
********
***************************************************
4861
AACTACCGCTTGTACTCAAGGAGCCACTTTGTAAAAGGGCTGGAGCTAATGGTATTGCTT

1003
ATATGTTATAGGCTATATGGTTCTGCAGCAGATGATGGTATCTCTTACGCACTCCTCTCA
******** *****
*********************************************
4921
ATATGTTACAGGCTGTATGGTTCTGCAGCAGATGATGGTATCTCTTACGCACTCCTCTCA

1063
TTTTCAATGTGGTTCTTAGTTTTATCCTGGTTGTTTGCTCCTTTCCTTCTGAATCCATCG
******************************
*****************************
4981
TTTTCAATGTGGTTCTTAGTTTTATCCTGGCTGTTTGCTCCTTTCCTTCTGAATCCATCG

1123
GGATTTGAATGGCAAAAGATAGTAGAAGATTGGGAAGACTGGTCAAAGTGGATAAGTTGC

************************************************************
5041
GGATTTGAATGGCAAAAGATAGTAGAAGATTGGGAAGACTGGTCAAAGTGGATAAGTTGC

1183
AGAGGTGGTATTGGAGTTCCCTCCGTTAAGAGCTGGGAATCTTGGTGGGAGGAAGAACAG

************************************************************
5101
AGAGGTGGTATTGGAGTTCCCTCCGTTAAGAGCTGGGAATCTTGGTGGGAGGAAGAACAG

1243
GAGCACCTGCGCCATACTGGATTTATAGGACGTTTCTTTGAGATTATACTTTCAATACGC
     ************** ******************
**************************
5161
GAGCACCTGCGCCACACTGGATTTATAGGACGTCTCTTTGAGATTATACTTTCAATACGC

1303
TTTTTATTTACCAGTATGGAATTGTGTATCATCTAAACATGACCACCAGTAGCAGACAA
     ** **
******************************************************
5221
TTCTTCATTTACCAGTATGGAATTGTGTATCATCTAAACATGACCACCAGTAGCAGACAA

1363
GGTATTCGGCTTAGCATTGTGGTTTATGGTCTTTCCTGGTTGGTCATTGGTGCTGTGTTG
     *********** ************************** *************
******
5281
GGTATTCGGCTGAGCATTGTGGTTTATGGTCTTTCCTGGCTGGTCATTGGTGCGGTGTTG

1423
ATTATTTTGAAGATAGTGTCGATGGGGAGAATGAAGTTCAGTGCGGATTTCCAGTTGATG
     ******************************************************
*****
5341
ATTATTTTGAAGATAGTGTCGATGGGGAGAATGAAGTTCAGTGCGGATTTCCAGCTGATG

1483
TTCAGACTTCTTAAGCTATTACTGTTTATTGGGTGTATAGTCACCATTGCAATGTTGTTT
     ****************** *******************
*********************
5401
TTCAGACTTCTTAAGCTACTACTGTTTATTGGGTGTATTGTCACCATTGCAATGTTGTTT

1543
TATTTCCTTAATCTCACAATTGGAGATATCTTCCAGAGCATACTGGCCTTTATGCCGACA
     ****************** *************
***************************
5461
TATTTCCTTAATCTCACAGTTGGAGATATCTTTCAGAGCATACTGGCCTTTATGCCGACA

```
1603
GGGTGGGCTCTTCTGCAGATATCACAAGCATGTCGAACACTGGTGAAGGGAATAGGAATG
     ******************************** ***
***********************
5521
GGGTGGGCTCTTCTGCAGATATCACAAGCATGCCGAGCACTGGTGAAGGGAATAGGAATG

1663
TGGGGGTCAGTAAAGGCACTAGCAAGAGGGTATGAATACATGATGGGTGTGTTACTGTTT
     ***********
************************************************
5581
TGGGGGTCAGTGAAGGCACTAGCAAGAGGGTATGAATACATGATGGGTGTGTTACTGTTT

1723
GCACCAATAGCTATATTGGCATGGTTCCCCTTCGTCTCAGAATTCCAGACCAGGCTGCTA

************************************************************
5641
GCACCAATAGCTATATTGGCATGGTTCCCCTTCGTCTCAGAATTCCAGACCAGGCTGCTA

1783
TTCAACCAAGCTTTCAGCCGAGGCCTCCAAATCCAACGTATTCTGGCTGGCAGCAAGAAG

************************************************************
5701
TTCAACCAAGCTTTCAGCCGAGGCCTCCAAATCCAACGTATTCTGGCTGGCAGCAAGAAG

1843  CAAGCCTAA
     *********
5761  CAAGCCTAA
```
