# Supplementary material for: GhCalS5 is involved in cotton response to aphid attack through mediating callose formation
Source: Front Plant Sci. 2022 Jul 20;13:892630. doi: 10.3389/fpls.2022.892630 (PMC9350506; doi:10.3389/fpls.2022.892630)
Supplement: Supplementary file 10 [file Table_2.docx]

Supplementary Material

| Gene | Forward primer sequence(5'-3') | Reverse primer sequence(5'-3') |
| --- | --- | --- |
| *GhCalS5* | CGCTCTAGAATGACATTTCGTGGAATATTTTCC | TGGATCCTTAGGCTTGCTTCTTGCTGC |
| *GhCalS5-RT* | ATGTGGGGGTCAGTAAAGGCACT | TTAGGCTTGCTTCTTGCTGCCAG |
| *GhUBI1* | CTGAATCTTCGCTTTCACGTTATC | GGGATGCAAATCTTCGTGAAAAC |
| *GhCalS5-like* | ATGAGGTCGTACCTTCCTCACTT | CTAACCTGTGTGCTTTCTCAAAAGC |
| *GhCalS 5.1* | ATGCATGGCATGAAATTTTCCCTAA | TTATCTCTCCAAGGCGATCA |

**Table S1** Sequences for primers that were used in this research.
