# Supplementary material for: GhCalS5 is involved in cotton response to aphid attack through mediating callose formation
Source: Front Plant Sci. 2022 Jul 20;13:892630. doi: 10.3389/fpls.2022.892630 (PMC9350506; doi:10.3389/fpls.2022.892630)
Supplement: Supplementary file 11 [file Table_3.DOCX]

Supplementary Material 1

***GhCalS5* ORF sequence: 1851bp**

ATGACATTTCGTGGCAATATTTTCCAGGACAATTACTTGGAAGAAGCATTCAAAATGCGTAATCTTTTGGAAGAATTTAATGAGGATCATGGAGTAAGGCCACCTACGATTTTAGGAGTTCGTGAGCACATCTTTACGGGAAGTGTTTCTTCTTTGGCTTGGTTCATGTCAAATCAAGAAACAAGCTTTGTCACCATTGGTCAAAGAGTTCTTGCAAGACCACTCAAGGTTCGCTTCCATTATGGTCATCCAGATGTGTTCGATAGAATCTTCCACATAACCCGTGGAGGCATCAGCAAGGGTTCTCGTGGCATCAACTTGAGTGAAGACATCTTTGCTGGTTTTAACTCAACCCTGAGACGAGGGAACATTACTCATCATGAATATATTCAGGTTGGGAAAGGTAGGGATGTTGGGTTAAACCAAATCTCACTTTTTGAAGCGAAAGTGGCTTGTGGTAACGGGGAGCAGACACTCAGCAGAGACATCTACAGATTAGGCCATCGTTTTGACTTTTTCCGCATGTTGTCCTGCTACTTTACCACTGTTGGATTTTATTTCAGCTCAATGTTGGTTGTCTTTACAGTCTACTTTTTCCTGTATGGAAGACTTTATTTGTCATTGAGTGGTTTAGAGGAGGCAATACTGAAGTATGCTTCAGCTAGGGGAAATAATTCTCTAAGGGCGGCCATGGCTTCACAGTCTATAGTTCAATTAGGTATCTTAACTGTACTACCCATGGTCATGGAGATTGGATTGGAGAGAGGATTTAGAACTGCATTAGGTGACATCATAATCATGCAGCTTCAGTTGGCATCCGTGTTCTTCACTTTCTCCCTTGGAACAAGAGTCCATTATTTTGGGCGCACTATTTTGCATGGTGGGGCTAAATACAGAGCAACAGGGCGTGGTTTTGTGGTGCGACATGAGAAATTCGCAGAGAACTACCGATTGTACTCAAGGAGCCACTTTGTAAAAGGGCTGGAGCTAATGGTATTGCTTATATGTTATAGGCTATATGGTTCTGCAGCAGATGATGGTATCTCTTACGCACTCCTCTCATTTTCAATGTGGTTCTTAGTTTTATCCTGGTTGTTTGCTCCTTTCCTTCTGAATCCATCGGGATTTGAATGGCAAAAGATAGTAGAAGATTGGGAAGACTGGTCAAAGTGGATAAGTTGCAGAGGTGGTATTGGAGTTCCCTCCGTTAAGAGCTGGGAATCTTGGTGGGAGGAAGAACAGGAGCACCTGCGCCATACTGGATTTATAGGACGTTTCTTTGAGATTATACTTTCAATACGCTTTTTTATTTACCAGTATGGAATTGTGTATCATCTAAACATGACCACCAGTAGCAGACAAGGTATTCGGCTTAGCATTGTGGTTTATGGTCTTTCCTGGTTGGTCATTGGTGCTGTGTTGATTATTTTGAAGATAGTGTCGATGGGGAGAATGAAGTTCAGTGCGGATTTCCAGTTGATGTTCAGACTTCTTAAGCTATTACTGTTTATTGGGTGTATAGTCACCATTGCAATGTTGTTTTATTTCCTTAATCTCACAATTGGAGATATCTTCCAGAGCATACTGGCCTTTATGCCGACAGGGTGGGCTCTTCTGCAGATATCACAAGCATGTCGAACACTGGTGAAGGGAATAGGAATGTGGGGGTCAGTAAAGGCACTAGCAAGAGGGTATGAATACATGATGGGTGTGTTACTGTTTGCACCAATAGCTATATTGGCATGGTTCCCCTTCGTCTCAGAATTCCAGACCAGGCTGCTATTCAACCAAGCTTTCAGCCGAGGCCTCCAAATCCAACGTATTCTGGCTGGCAGCAAGAAGCAAGCCTAA

***GhCalS5* Amino acid sequence: 616 aa**

MTFRGNIFQDNYLEEAFKMRNLLEEFNEDHGVRPPTILGVREHIFTGSVSSLAWFMSNQETSFVTIGQRVLARPLKVRFHYGHPDVFDRIFHITRGGISKGSRGINLSEDIFAGFNSTLRRGNITHHEYIQVGKGRDVGLNQISLFEAKVACGNGEQTLSRDIYRLGHRFDFFRMLSCYFTTVGFYFSSMLVVFTVYFFLYGRLYLSLSGLEEAILKYASARGNNSLRAAMASQSIVQLGILTVLPMVMEIGLERGFRTALGDIIIMQLQLASVFFTFSLGTRVHYFGRTILHGGAKYRATGRGFVVRHEKFAENYRLYSRSHFVKGLELMVLLICYRLYGSAADDGISYALLSFSMWFLVLSWLFAPFLLNPSGFEWQKIVEDWEDWSKWISCRGGIGVPSVKSWESWWEEEQEHLRHTGFIGRFFEIILSIRFFIYQYGIVYHLNMTTSSRQGIRLSIVVYGLSWLVIGAVLIILKIVSMGRMKFSADFQLMFRLLKLLLFIGCIVTIAMLFYFLNLTIGDIFQSILAFMPTGWALLQISQACRTLVKGIGMWGSVKALARGYEYMMGVLLFAPIAILAWFPFVSEFQTRLLFNQAFSRGLQIQRILAGSKKQA

**File S1** *GhCalS5* and amino acid sequence.
