# Supplementary material for: GhCalS5 is involved in cotton response to aphid attack through mediating callose formation
Source: Front Plant Sci. 2022 Jul 20;13:892630. doi: 10.3389/fpls.2022.892630 (PMC9350506; doi:10.3389/fpls.2022.892630)
Supplement: Supplementary file 1 [file Data_Sheet_1.PDF]

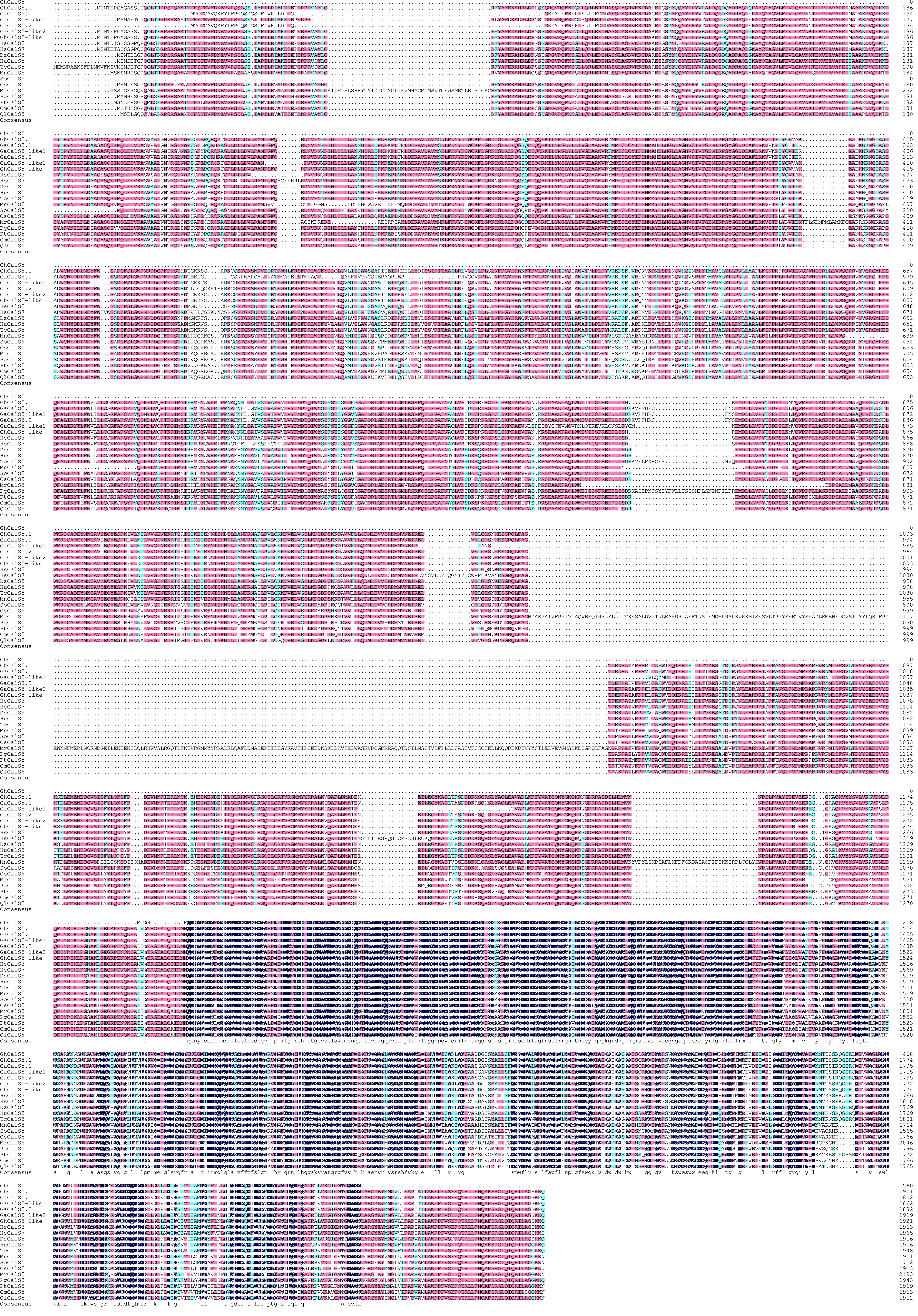
GhCalS5
GhCalS5.1
GaCalS5.1
GaCalS5-like1
GaCalS5.2
GaCalS5-like2
GhCalS5-like
HsCalS3
HsCalS7
DzCalS5
HuCalS5
TcCalS5
MnCalS5
SoCalS5
CsCalS5
MrCalS5
PgCalS5
PtCalS5
CmCalS5
QlCalS5
Consensus
